# Crystal structures of *trans*-di­chlorido­tetra­kis­[1-(2,6-diiso­propyl­phen­yl)-1*H*-imidazole-κ*N* ^3^]iron(II), *trans*-di­bromido­tetra­kis­[1-(2,6-diiso­propyl­phen­yl)-1*H*-imidazole-κ*N* ^3^]iron(II) and *trans*-di­bromido­tetra­kis­[1-(2,6-diiso­propyl­phen­yl)-1*H*-imidazole-κ*N* ^3^]iron(II) diethyl ether disolvate^1^

**DOI:** 10.1107/S1600536814014056

**Published:** 2014-07-19

**Authors:** Roger Mafua, Titus Jenny, Gael Labat, Antonia Neels, Helen Stoeckli-Evans

**Affiliations:** a Department of Chemistry, University of Fribourg, Av. de Perolles, CH-1700 Fribourg, Switzerland; b Benefri Crystallography Service, University of Neuchâtel, Av. de Bellvaux 51, CH-2000 Neuchâtel, Switzerland; c Institute of Physics, University of Neuchâtel, rue Emile-Argand 11, CH-2000 Neuchâtel, Switzerland

**Keywords:** aryl­imidazole, iron(II), crystal structure

## Abstract

The title compounds, [FeCl_2_(C_15_H_20_N_2_)_4_], (I), [FeBr_2_(C_15_H_20_N_2_)_4_], (II), and [FeBr_2_(C_15_H_20_N_2_)_4_]·2C_4_H_10_O, (II*b*), respectively, all have triclinic symmetry, with (I) and (II) being isotypic. The Fe^II^ atoms in each of the structures are located on an inversion center. They have octa­hedral Fe*X*_2_N_4_ (*X* = Cl and Br, respectively) coordination spheres with the Fe^II^ atom coordinated by two halide ions in a *trans* arrangement and by the tertiary N atom of four aryl­imidazole ligands [1-(2,6-diiso­propyl­phen­yl)-1*H*-imidazole] in the equatorial plane. In the two independent ligands, the benzene and imidazole rings are almost normal to one another, with dihedral angles of 88.19 (15) and 79.26 (14)° in (I), 87.0 (3) and 79.2 (3)° in (II), and 84.71 (11) and 80.58 (13)° in (II*b*). The imidazole rings of the two independent ligand mol­ecules are inclined to one another by 70.04 (15), 69.3 (3) and 61.55 (12)° in (I), (II) and (II*b*), respectively, while the benzene rings are inclined to one another by 82.83 (13), 83.0 (2) and 88.16 (12)°, respectively. The various dihedral angles involving (II*b*) differ slightly from those in (I) and (II), probably due to the close proximity of the diethyl ether solvent mol­ecule. There are a number of C—H⋯halide hydrogen bonds in each mol­ecule involving the CH groups of the imidazole units. In the structures of compounds (I) and (II), mol­ecules are linked *via* pairs of C—H⋯halogen hydrogen bonds, forming chains along the *a* axis that enclose *R*_2_^2^(12) ring motifs. The chains are linked by C—H⋯π inter­actions, forming sheets parallel to (001). In the structure of compound (II*b*), mol­ecules are linked *via* pairs of C—H⋯halogen hydrogen bonds, forming chains along the *b* axis, and the diethyl ether solvent mol­ecules are attached to the chains *via* C—H⋯O hydrogen bonds. The chains are linked by C—H⋯π inter­actions, forming sheets parallel to (001). In (I) and (II), the methyl groups of an isopropyl group are disordered over two positions [occupancy ratio = 0.727 (13):0.273 (13) and 0.5:0.5, respectively]. In (II*b*), one of the ethyl groups of the diethyl ether solvent mol­ecule is disordered over two positions (occupancy ratio = 0.5:0.5).

## Abstract 2

The title compounds are iron(II) dihalide complexes of the bulky arylimidazole ligand 1-(2,6-diisopropylphenyl)-1*H*-imidazole. The FeCl_2_ and FeBr_2_ complexes are isotypic, while the third compound, also an FeBr_2_ complex, crystallizes as a diethyl ether disolvate.

## Chemical context   

The use of organometallic complexes as catalysts is an important development in the field of material chemistry. However, despite this, only a very few of them contain iron(II), except the tridentate di­imine pyridine complex (Small *et al.*, 1998 ▸; Small & Brookhart, 1998 ▸; Britovsek *et al.*, 1998 ▸) used in olefin polymerization. Unfortunately, this model suffers from its lack of tolerance towards the minor changes carried out in its envelope, resulting in a drastic reduction of its catalytic activity. Neutral and cationic complexes of iron(II) chloride and bromide with nitro­gen bases are well known for imidazole, pyridine and pyrazoles (Schröder *et al.*, 2009 ▸; Christie *et al.*, 1993 ▸). For this reason, we set out to prepare new iron complexes containing more electron-donating and bulky ligands. Only a few analogous bulky aryl­imidazoles have been reported so far (Reisner *et al.*, 2007 ▸).
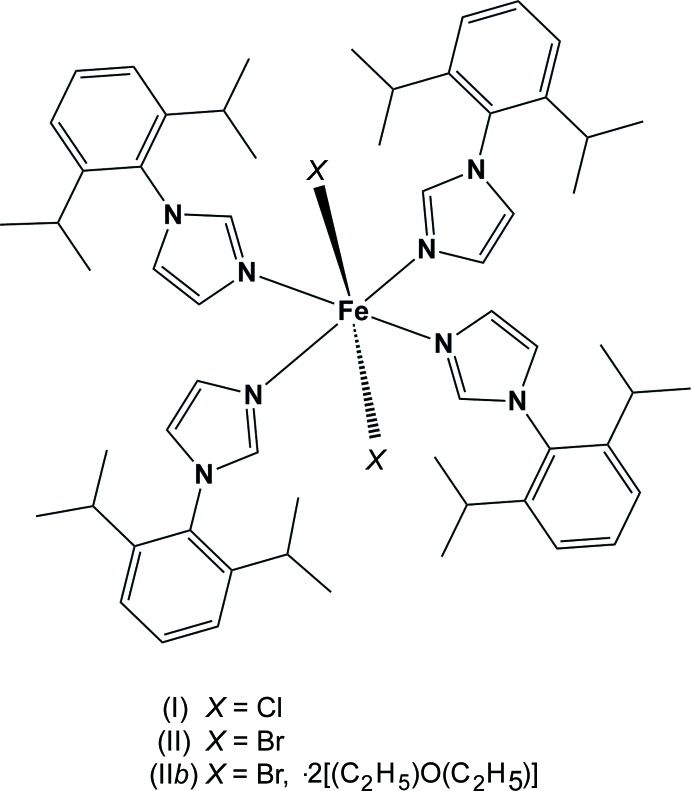


We focused our attention on the use of bis-*N*-heterocyclic carbene Fe^II^ complexes in hydrogenation and polymerization of olefins (Mafua, 2006 ▸). During the preparation of these complexes, several other complexes of Fe^II^ and Fe^III^ were isolated, among them the title compounds, (I), (II) and (II*b*). Compound (I) was isolated by deprotonation of bis­imidazoliummethyl­ene tetra­chlorido­ferrate(III) (*L*1 in Fig. 7 ▸) with NaH in THF at reflux. When the same reaction was conducted at room temperature, only the starting material was recovered after recrystallization. Compounds (II) and (II*b*) were isolated when bis­imidazoliummethyl­ene tetra­bromido­ferrate(III) (*L*2 in Fig. 7 ▸) was reacted with NaH in THF at reflux. The main result in the structure of these compounds is the loss of the bridging methyl­ene group of the starting bis­imidazolium cation. Thus two independent N-1-aryl­imidazolyl groups are formed for each starting bis­imidazolium cation. Additionally, this result demonstrates a possible fragility of methyl­ene-bis­imidazole ligands when used in harsh reaction conditions. The question of the reduction of Fe^III^ to Fe^II^ remains to be elucidated.

## Structural commentary   

**Figure 1:**
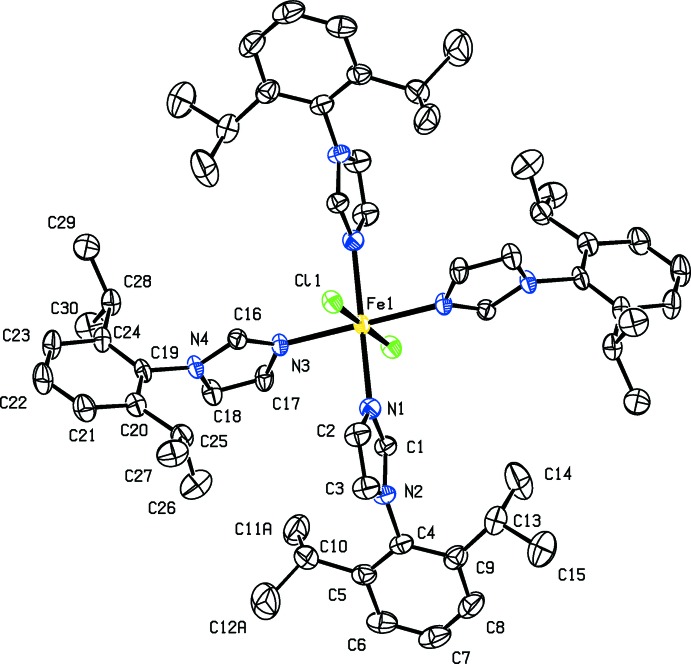
A view of the mol­ecular structure of complex (I), with atom labelling. Displacement ellipsoids are drawn at the 50% probability level; disordered parts are not shown. H atoms have been omitted for clarity.

The structures of (I) and (II) are isotypic whereas (II*b*) differs due to the presence of solvent diethyl ether mol­ecules. The whole mol­ecule of each compound, (I), (II) and (II*b*), is generated by inversion symmetry (Figs. 1 ▸, 2 ▸ and 3 ▸, respectively). The Fe^II^ atom, Fe1, is located on an inversion center and has an octa­hedral Fe*X*_2_N_4_ (*X* = Br, Cl) coordination sphere. It is coordinated by the tertiary N atoms of four imidazole ligands [1-(2,6-diiso­propyl­phen­yl)-1*H*-imidazole], in the equatorial plane, while the axial positions are occupied by the halogen ions. In (I), the axial Fe1—Cl1 bond length is 2.5356 (9) Å, while the equatorial Fe1—N1 and Fe1—N3 bond lengths are 2.188 (2) and 2.161 (2) Å, respectively. In the structures of compounds (II) and (II*b*), the Fe—Br1 bond lengths are 2.7040 (5) and 2.7422 (3) Å, respectively. The Fe—N1 and Fe1—N3 bond lengths are 2.190 (3) and 2.161 (3) Å in (II), and 2.1889 (16) and 2.1789 (15) Å in (II*b*). In each mol­ecule, all of the imidazole C-bound H atoms are involved in intra­molecular C—H⋯halogen hydrogen bonds (see Tables 1 ▸, 2 ▸ and 3 ▸).

In the two independent ligands of (I), the benzene rings (C4–C9 and C19–C24) are inclined to their attached imidazole rings (N1/N2/C1–C3 and N3/N4/C16–C18, respectively) by 88.19 (15) and 79.26 (14)°. In (II) and (II*b*), the corresponding angles are 87.0 (3) and 79.2 (3)°, and 84.71 (11) and 80.58 (13)°, respectively. The imidazole rings (N1/N2/C1–C3 and N3/N4/C16-C18) of the two independent ligand mol­ecules are inclined to one another by 70.04 (15), 69.3 (3) and 61.55 (12)° in (I), (II) and (II*b*), respectively, while the benzene rings (C4–C9 and C19–C24) are inclined to one another by 82.83 (13), 83.0 (2) and 88.16 (12)°, respectively. The various dihedral angles involving (II*b*) differ slightly from those in (I) and (II) due to steric hindrance owing to the close proximity of the diethyl ether solvent mol­ecule of crystallization.

## Supra­molecular features   

**Figure 4:**
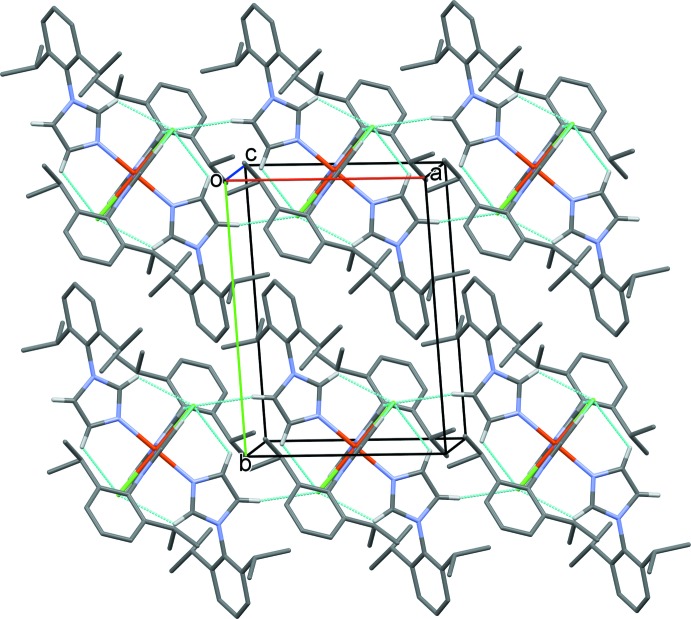
A view along the *c* axis of the crystal packing of compound (I). Hydrogen bonds are shown as dashed lines (see Table 1 ▸ for details; H atoms not involved in these inter­actions have been omitted for clarity).

**Figure 6:**
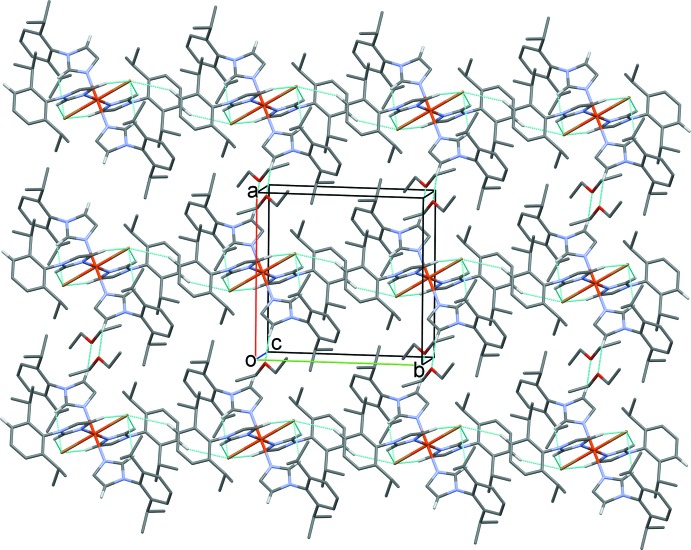
A view along the *c* axis of the crystal packing of compound (II*b*). Hydrogen bonds are shown as dashed lines (see Table 3 ▸ for details; H atoms not involved in these inter­actions have been omitted for clarity).

In the crystal structures of all three compounds, (I), (II) and (II*b*), mol­ecules are linked *via* pairs of C—H⋯halogen hydrogen bonds, forming chains along the *a* axis [for (I) and (II)] and the *b* axis, respectively, for (II*b*) that enclose 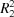
(12) ring motifs (Figs. 4 ▸, 5 ▸ and 6 ▸, respectively, and Tables 1 ▸, 2 ▸ and 3 ▸, respectively). They are linked by C—H⋯π inter­actions, forming sheets parallel to (001). In the crystal structure of compound (II*b*), the diethyl ether solvent mol­ecules are attached to the chains *via* C—H⋯O hydrogen bonds, and within the chains there are a series of C—H⋯π inter­actions present (Fig. 6 ▸ and Table 3 ▸).

## Database survey   

A search of the Cambridge Structural Database (Version 5.35, last update November 2013; Allen, 2002 ▸) indicated the presence of five tetra­kis­(*N*-substituted imidazole) iron halide complexes. Two of these involve iron(II), that is *trans*-dichlorido­tetra­kis­(5-chloro-1-methyl-1*H*-imidazole-*N*-iron(III) chloride hydrate (Schröder *et al.*, 2009 ▸) and *trans*-di­fluoridotetra­kis­(1-methyl­imidazole)­iron(III) tetra­fluorido­borate (Chris­tie *et al.*, 1993 ▸). Two compounds containing aryl-substituted imidazoles where found, namely (μ_2_-oxido)-tetra­chlorido­tetra­kis­(1-phenyl-1*H*-imidazole-*N*)diiron(II) and (μ_2_-oxido)tetra­chlorido­tetra­kis­[1-(2,6-disio­propyl­phen­yl)-1*H*-imidazole-*N*]diiron(II) (Schröder *et al.*, 2009 ▸). The crystal structure of di­chlorido­tetra­kis­(1-methyl­imidazole-*N* ^3^)iron(II) has also been reported (Reisner *et al.*, 2007 ▸).

## Synthesis and crystallization   

**Figure 7:**
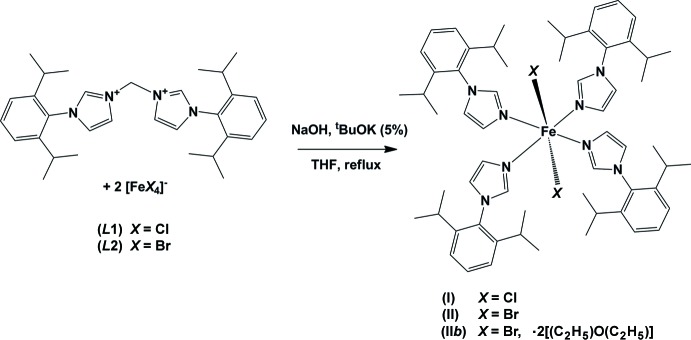
Reaction scheme.

The synthesis of the precursors bis­imidazolium methyl­ene tetra­chlorido- and tetra­bromido­ferrate(III) (*L*1 and *L*2, respectively, in Fig. 7 ▸) have been reported elsewhere (Mafua, 2006 ▸). Compound (I) was prepared as follows: to a solution of (*L*1) [0.34 g, 0.5 mmol] in 20 ml of THF was added 0.09 g (2.3 mmol) of NaH 60% and 0.01 g (0.1 mmol) of *^t^*BuOK, and the reaction mixture was heated at 340 K for 8 h. The solution was then filtered and the solvent evaporated under vacuum yielding an orange solid. Yellow crystals were obtained by slow diffusion of diethyl ether into a THF solution of the isolated orange solid. UV–vis (THF, 200–800 nm): 364, 290. Compounds (II) and (II*b*) were prepared in a similar manner. To a solution of (*L*2) [0.29 g, 0.5 mmol] in 20 ml of THF was added 0.09 g (2.3 mmol) of NaH 60% and 0.01 g (0.1 mmol) of *^t^*BuOK at 273 K, and the reaction mixture was heated at reflux for 8 h. The solution was then filtered and the solvent evaporated under vacuum yielding a yellow–brown solid. Yellow crystals were obtained by slow diffusion of diethyl ether into a THF solution of the isolated yellow–brownish solid. UV–vis (THF, 200–800 nm): 292. Two types of crystals were obtained: yellow plates for (II) and yellow blocks for (II*b*).

## Refinement   

Crystal data, data collection and structure refinement details are summarized in Table 4 ▸. In all three compounds, the H atoms were included in calculated positions and treated as riding atoms: C—H = 0.95, 1.00 and 0.98 Å for CH(aromatic), CH and CH_3_ H atoms, respectively, with *U*_iso_(H) = 1.5*U*_eq_(C-meth­yl) and = 1.2*U*_eq_(C) for other H atoms. In (I) and (II), the methyl groups of an isopropyl group are disordered over two positions [occupancy ratio = 0.727 (13):0.273 (13) in (I) and fixed at 0.5:0.5 for (II)]. In (II*b*), one of the ethyl groups of the diethyl ether solvent mol­ecule is disordered over two positions (occupancy ratio fixed at 0.5:0.5).

## Supplementary Material

Crystal structure: contains datablock(s) I, II, IIb, Global. DOI: 10.1107/S1600536814014056/wm0006sup1.cif

Structure factors: contains datablock(s) I. DOI: 10.1107/S1600536814014056/wm0006Isup2.hkl

Structure factors: contains datablock(s) II. DOI: 10.1107/S1600536814014056/wm0006IIsup3.hkl

Structure factors: contains datablock(s) IIb. DOI: 10.1107/S1600536814014056/wm0006IIbsup4.hkl

CCDC references: 1008173, 1008174, 1008175

Additional supporting information:  crystallographic information; 3D view; checkCIF report

## Figures and Tables

**Figure 2:**
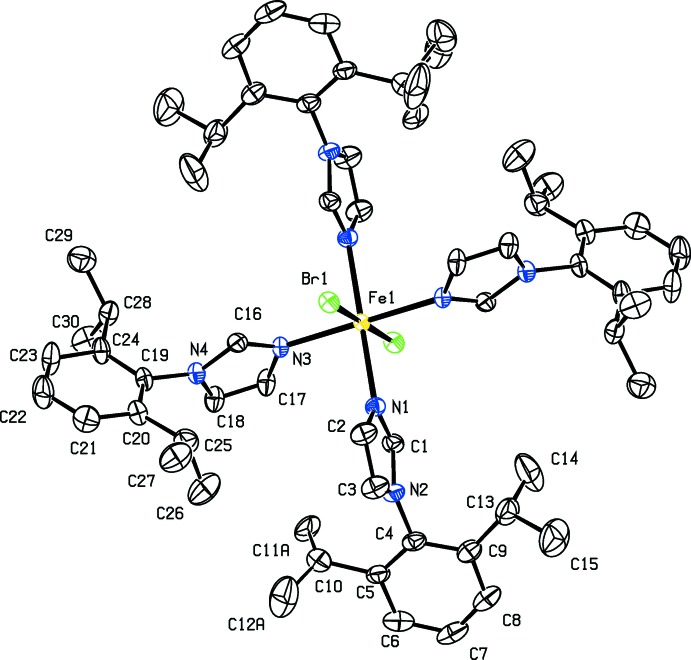
A view of the mol­ecular structure of complex (II), with atom labelling. Displacement ellipsoids are drawn at the 50% probability level; disordered parts are not shown. H atoms have been omitted for clarity.

**Figure 3:**
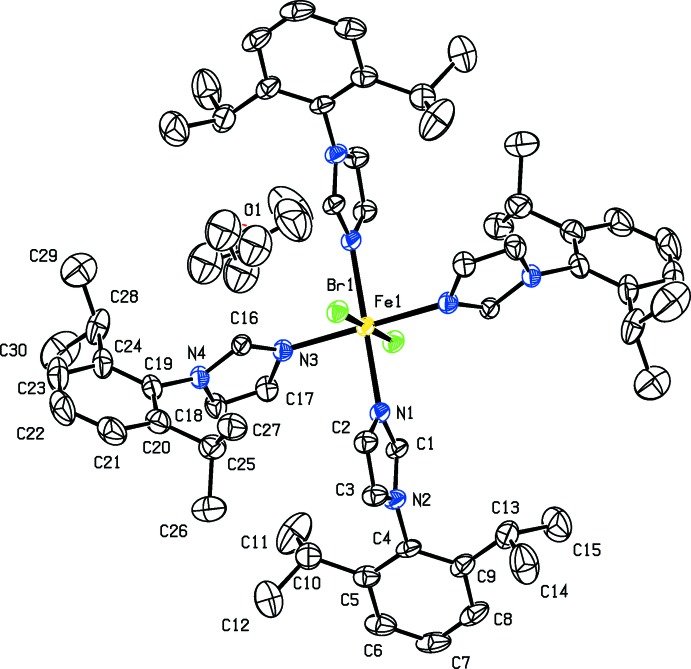
A view of the mol­ecular structure of complex (II*b*), with atom labelling. Displacement ellipsoids are drawn at the 50% probability level; disordered parts are not shown. H atoms have been omitted for clarity.

**Figure 5:**
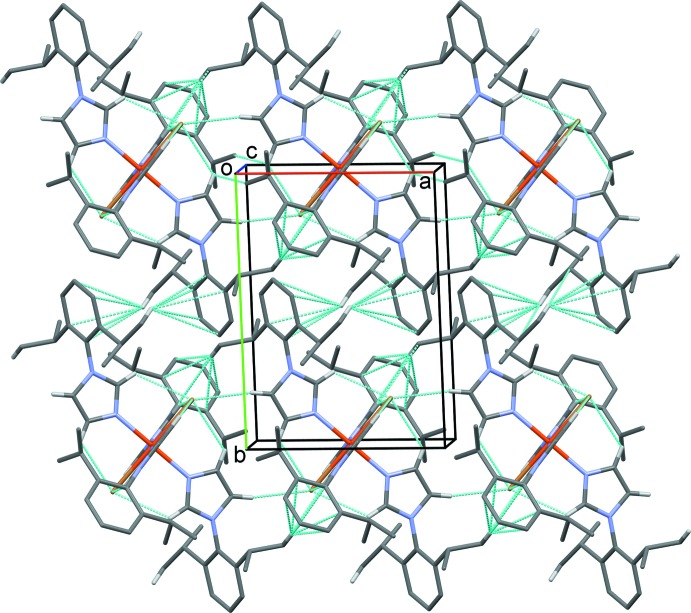
A view along the *c* axis of the crystal packing of compound (II). Hydrogen bonds and C—H⋯π inter­actions are shown as dashed lines (see Table 2 ▸ for details; H atoms not involved in these inter­actions have been omitted for clarity).

**Table 1:** Hydrogen-bond geometry (Å, °) for (I) *Cg*3 and *Cg*4 are the centroids of rings C4–C9 and C19–C24, respectively.

*D*—H⋯*A*	*D*—H	H⋯*A*	*D*⋯*A*	*D*—H⋯*A*
C1—H1⋯Cl1^i^	0.95	2.62	3.257 (3)	125
C2—H2⋯Cl1	0.95	2.92	3.433 (3)	115
C16—H16⋯Cl1	0.95	2.76	3.294 (3)	117
C17—H17⋯Cl1^i^	0.95	2.82	3.375 (3)	118
C18—H18⋯Cl1^ii^	0.95	2.70	3.629 (3)	166
C27—H27*A*⋯*Cg*4^iii^	0.98	2.79	3.562 (4)	136
C30—H30*C*⋯*Cg*3^iv^	0.98	2.92	3.901 (4)	176

Symmetry codes: (i) 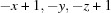; (ii) 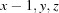; (iii) 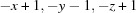; (iv) 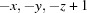.

**Table 2:** Hydrogen-bond geometry (Å, °) for (II) *Cg*3 and *Cg*4 are the centroids of rings C4–C9 and C19–C24, respectively.

*D*—H⋯*A*	*D*—H	H⋯*A*	*D*⋯*A*	*D*—H⋯*A*
C1—H1⋯Br1^i^	0.95	2.71	3.368 (4)	127
C2—H2⋯Br1	0.95	2.91	3.477 (5)	119
C16—H16⋯Br1	0.95	2.81	3.373 (4)	119
C17—H17⋯Br1^i^	0.95	2.91	3.484 (4)	120
C18—H18⋯Br1^ii^	0.95	2.77	3.707 (5)	167
C27—H27*A*⋯*Cg*4^iii^	0.98	2.92	3.639 (6)	131
C30—H30*C*⋯*Cg*3^iv^	0.98	2.88	3.862 (6)	177

Symmetry codes: (i) 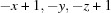; (ii) 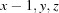; (iii) 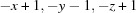; (iv) 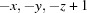.

**Table 3:** Hydrogen-bond geometry (Å, °) for (II*b*) *Cg*2 and *Cg*3 are the centroids of rings N3/N4/C16–C18 and C4–C9, respectively.

*D*—H⋯*A*	*D*—H	H⋯*A*	*D*⋯*A*	*D*—H⋯*A*
C1—H1⋯Br1^i^	0.95	2.76	3.399 (2)	125
C2—H2⋯Br1	0.95	2.89	3.479 (2)	121
C16—H16⋯Br1	0.95	2.86	3.4119 (18)	118
C17—H17⋯Br1^i^	0.95	3.02	3.542 (2)	116
C18—H18⋯O1^ii^	0.95	2.40	3.337 (3)	170
C15—H15*A*⋯*Cg*3^iii^	0.98	2.92	3.801 (3)	150
C25—H25⋯*Cg*2	1.00	2.61	3.413 (2)	137
C26—H26*A*⋯*Cg*3^iv^	0.98	2.87	3.682 (3)	140
C34*B*—H34*E*⋯*Cg*2^v^	0.98	2.92	3.627 (9)	130

Symmetry codes: (i) 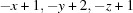; (ii) 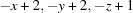; (iii) 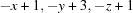; (iv) 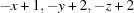; (v) 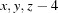.

**Table 4:** Experimental details

	(I)	(II)	(II*b*)
Crystal data			
Chemical formula	[FeCl_2_(C_15_H_20_N_2_)_4_]	[FeBr_2_(C_15_H_20_N_2_)_4_]	[FeBr_2_(C_15_H_20_N_2_)_4_]·2C_4_H_10_O
*M*_r_	1040.07	1128.99	1277.22
Crystal system, space group	Triclinic, *P* 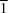	Triclinic, *P* 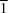	Triclinic, *P* 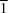
Temperature (K)	173	173	173
*a*, *b*, *c* (Å)	8.877 (2), 12.628 (3), 13.810 (4)	9.0391 (11), 12.7658 (11), 13.689 (2)	11.6710 (8), 12.4758 (9), 13.5759 (10)
α, β, γ (°)	74.68 (2), 74.48 (2), 83.105 (18)	74.502 (9), 74.481 (12), 84.343 (9)	64.464 (5), 81.515 (6), 88.982 (6)
*V* (Å^3^)	1436.6 (7)	1466.0 (3)	1761.8 (2)
*Z*	1	1	1
Radiation type	Mo *K*α	Mo *K*α	Mo *K*α
μ (mm^−1^)	0.40	1.66	1.39
Crystal size (mm)	0.25 × 0.20 × 0.15	0.20 × 0.17 × 0.10	0.50 × 0.50 × 0.50

Data collection			
Diffractometer	Stoe *IPDS* 2	Stoe *IPDS* 2	Stoe *IPDS* 2
Absorption correction	Multi-scan (*MULscanABS* in *PLATON*; Spek, 2009 ▸)	Multi-scan (*MULscanABS* in *PLATON*; Spek, 2009 ▸)	Multi-scan (*MULscanABS* in *PLATON*; Spek, 2009 ▸)
*T*_min_, *T*_max_	0.966, 1.000	0.457, 0.496	0.557, 0.672
No. of measured, independent and observed [*I* > 2σ(*I*)] reflections	14618, 5214, 3012	17613, 5312, 3013	15799, 6374, 5714
*R*_int_	0.082	0.118	0.030
(sin θ/λ)_max_ (Å^−1^)	0.600	0.600	0.600

Refinement			
*R*[*F* ^2^ > 2σ(*F* ^2^)], *wR*(*F* ^2^), *S*	0.043, 0.069, 0.80	0.046, 0.081, 0.81	0.031, 0.077, 1.03
No. of reflections	5214	5312	6374
No. of parameters	339	350	378
No. of restraints	4	2	0
H-atom treatment	H-atom parameters constrained	H-atom parameters constrained	H-atom parameters constrained
Δρ_max_, Δρ_min_ (e Å^−3^)	0.23, −0.19	0.59, −0.64	0.44, −0.37

Computer programs: *X-AREA* and *X-RED32* (Stoe & Cie, 2006 ▸), *SHELXS97* and *SHELXL2013* (Sheldrick, 2008 ▸), *PLATON* (Spek, 2009 ▸), *Mercury* (Macrae *et al.*, 2008 ▸) and *publCIF* (Westrip, 2010 ▸).

## supplementary crystallographic information

### (I) trans-Dichloridotetrakis[1-(2,6-diisopropylphenyl)-1H-imidazole-κN3]iron(II) Crystal data

**Table:**

[FeCl_2_(C_15_H_20_N_2_)_4_]	*Z* = 1
*M**_r_* = 1040.07	*F*(000) = 556
Triclinic, *P*1	*D*_x_ = 1.202 Mg m^−^^3^
Hall symbol: -P 1	Mo *K*α radiation, λ = 0.71073 Å
*a* = 8.877 (2) Å	Cell parameters from 7147 reflections
*b* = 12.628 (3) Å	θ = 0.1–24.9°
*c* = 13.810 (4) Å	µ = 0.40 mm^−^^1^
α = 74.68 (2)°	*T* = 173 K
β = 74.48 (2)°	Block, colourless
γ = 83.105 (18)°	0.25 × 0.20 × 0.15 mm
*V* = 1436.6 (7) Å^3^	

### (I) trans-Dichloridotetrakis[1-(2,6-diisopropylphenyl)-1H-imidazole-κN3]iron(II) Data collection

**Table:**

Stoe IPDS 2 diffractometer	5214 independent reflections
Radiation source: fine-focus sealed tube	3012 reflections with *I* > 2σ(*I*)
Plane graphite monochromator	*R*_int_ = 0.082
φ + ω scans	θ_max_ = 25.3°, θ_min_ = 1.6°
Absorption correction: multi-scan (MULscanABS in *PLATON*; Spek, 2009)	*h* = −10→10
*T*_min_ = 0.966, *T*_max_ = 1.000	*k* = −15→15
14618 measured reflections	*l* = −16→16

### (I) trans-Dichloridotetrakis[1-(2,6-diisopropylphenyl)-1H-imidazole-κN3]iron(II) Refinement

**Table:**

Refinement on *F*^2^	Primary atom site location: structure-invariant direct methods
Least-squares matrix: full	Secondary atom site location: difference Fourier map
*R*[*F*^2^ > 2σ(*F*^2^)] = 0.043	Hydrogen site location: inferred from neighbouring sites
*wR*(*F*^2^) = 0.069	H-atom parameters constrained
*S* = 0.80	*w* = 1/[σ^2^(*F*_o_^2^) + (0.0176*P*)^2^] where *P* = (*F*_o_^2^ + 2*F*_c_^2^)/3
5214 reflections	(Δ/σ)_max_ = 0.001
339 parameters	Δρ_max_ = 0.23 e Å^−^^3^
4 restraints	Δρ_min_ = −0.19 e Å^−^^3^

### (I) trans-Dichloridotetrakis[1-(2,6-diisopropylphenyl)-1H-imidazole-κN3]iron(II) Special details

**Table:**

Geometry. All e.s.d.'s (except the e.s.d. in the dihedral angle between two l.s. planes) are estimated using the full covariance matrix. The cell e.s.d.'s are taken into account individually in the estimation of e.s.d.'s in distances, angles and torsion angles; correlations between e.s.d.'s in cell parameters are only used when they are defined by crystal symmetry. An approximate (isotropic) treatment of cell e.s.d.'s is used for estimating e.s.d.'s involving l.s. planes.

### (I) trans-Dichloridotetrakis[1-(2,6-diisopropylphenyl)-1H-imidazole-κN3]iron(II) Fractional atomic coordinates and isotropic or equivalent isotropic displacement parameters (Å^2^)

**Table:**

	*x*	*y*	*z*	*U*_iso_*/*U*_eq_	Occ. (<1)
Fe1	0.5000	0.0000	0.5000	0.02088 (16)	
Cl1	0.70366 (8)	−0.15905 (5)	0.50114 (5)	0.02791 (18)	
N1	0.5146 (2)	0.01056 (16)	0.33706 (16)	0.0268 (5)	
N2	0.4532 (3)	0.06854 (17)	0.18661 (15)	0.0276 (5)	
N3	0.3206 (2)	−0.11518 (16)	0.53380 (15)	0.0242 (5)	
N4	0.2109 (2)	−0.26733 (16)	0.54788 (16)	0.0253 (5)	
C1	0.4309 (3)	0.0828 (2)	0.28262 (19)	0.0272 (7)	
H1	0.3626	0.1386	0.3081	0.033*	
C2	0.5931 (3)	−0.0546 (2)	0.2729 (2)	0.0353 (7)	
H2	0.6631	−0.1152	0.2910	0.042*	
C3	0.5573 (3)	−0.0208 (2)	0.1802 (2)	0.0364 (7)	
H3	0.5960	−0.0522	0.1223	0.044*	
C4	0.3853 (3)	0.1369 (2)	0.10628 (19)	0.0287 (7)	
C5	0.2450 (3)	0.1095 (2)	0.09679 (19)	0.0326 (7)	
C6	0.1819 (4)	0.1781 (2)	0.0180 (2)	0.0435 (8)	
H6	0.0857	0.1619	0.0087	0.052*	
C7	0.2585 (4)	0.2685 (3)	−0.0459 (2)	0.0500 (9)	
H7	0.2158	0.3134	−0.1001	0.060*	
C8	0.3955 (4)	0.2953 (2)	−0.0330 (2)	0.0474 (8)	
H8	0.4447	0.3595	−0.0772	0.057*	
C9	0.4638 (3)	0.2302 (2)	0.0439 (2)	0.0355 (7)	
C10	0.1572 (4)	0.0119 (2)	0.1701 (2)	0.0423 (8)	
H10A	0.2349	−0.0377	0.2039	0.051*	0.727 (13)
H10B	0.2081	−0.0198	0.2287	0.051*	0.273 (13)
C11A	0.0312 (9)	0.0456 (5)	0.2559 (6)	0.0590 (19)	0.727 (13)
H11A	0.0779	0.0835	0.2933	0.088*	0.727 (13)
H11B	−0.0186	−0.0199	0.3039	0.088*	0.727 (13)
H11C	−0.0475	0.0951	0.2262	0.088*	0.727 (13)
C12A	0.0892 (13)	−0.0560 (8)	0.1187 (8)	0.078 (3)	0.727 (13)
H12A	0.0538	−0.1247	0.1687	0.117*	0.727 (13)
H12B	0.1694	−0.0728	0.0598	0.117*	0.727 (13)
H12C	0.0002	−0.0146	0.0944	0.117*	0.727 (13)
C11B	−0.0112 (19)	0.0578 (14)	0.2122 (15)	0.0590 (19)	0.273 (13)
H11D	−0.0654	0.0800	0.1562	0.088*	0.273 (13)
H11E	−0.0058	0.1216	0.2387	0.088*	0.273 (13)
H11F	−0.0687	0.0008	0.2682	0.088*	0.273 (13)
C12B	0.159 (4)	−0.073 (2)	0.112 (3)	0.078 (3)	0.273 (13)
H12D	0.1249	−0.0384	0.0489	0.117*	0.273 (13)
H12E	0.0880	−0.1302	0.1559	0.117*	0.273 (13)
H12F	0.2656	−0.1056	0.0946	0.117*	0.273 (13)
C13	0.6097 (4)	0.2615 (2)	0.0631 (2)	0.0444 (8)	
H13	0.6631	0.1923	0.0958	0.053*	
C14	0.5694 (4)	0.3354 (3)	0.1394 (3)	0.0716 (11)	
H14A	0.5236	0.4063	0.1075	0.086*	
H14B	0.6648	0.3473	0.1572	0.086*	
H14C	0.4939	0.3000	0.2024	0.086*	
C15	0.7269 (4)	0.3182 (3)	−0.0346 (3)	0.0669 (11)	
H15A	0.7469	0.2747	−0.0863	0.080*	
H15B	0.8251	0.3247	−0.0174	0.080*	
H15C	0.6839	0.3917	−0.0626	0.080*	
C16	0.3362 (3)	−0.2225 (2)	0.55469 (18)	0.0253 (6)	
H16	0.4257	−0.2639	0.5726	0.030*	
C17	0.1766 (3)	−0.0894 (2)	0.5126 (2)	0.0301 (7)	
H17	0.1321	−0.0168	0.4946	0.036*	
C18	0.1064 (3)	−0.1826 (2)	0.5210 (2)	0.0308 (7)	
H18	0.0063	−0.1879	0.5105	0.037*	
C19	0.1907 (3)	−0.38269 (19)	0.5634 (2)	0.0259 (6)	
C20	0.2662 (3)	−0.4342 (2)	0.4836 (2)	0.0309 (7)	
C21	0.2407 (3)	−0.5445 (2)	0.5010 (2)	0.0385 (7)	
H21	0.2882	−0.5822	0.4484	0.046*	
C22	0.1479 (4)	−0.6007 (2)	0.5928 (2)	0.0450 (8)	
H22	0.1314	−0.6762	0.6027	0.054*	
C23	0.0796 (3)	−0.5482 (2)	0.6698 (2)	0.0381 (7)	
H23	0.0175	−0.5885	0.7332	0.046*	
C24	0.0987 (3)	−0.4372 (2)	0.6576 (2)	0.0292 (6)	
C25	0.3709 (3)	−0.3722 (2)	0.3836 (2)	0.0350 (7)	
H25	0.4332	−0.3231	0.4023	0.042*	
C26	0.2778 (4)	−0.2999 (3)	0.3121 (2)	0.0568 (10)	
H26A	0.2138	−0.3454	0.2931	0.068*	
H26B	0.3494	−0.2599	0.2494	0.068*	
H26C	0.2095	−0.2472	0.3472	0.068*	
C27	0.4861 (4)	−0.4467 (3)	0.3262 (2)	0.0492 (9)	
H27C	0.5622	−0.4022	0.2696	0.059*	
H27B	0.4297	−0.4885	0.2979	0.059*	
H27A	0.5410	−0.4977	0.3741	0.059*	
C28	0.0214 (3)	−0.3808 (2)	0.7438 (2)	0.0333 (7)	
H28	0.0714	−0.3092	0.7274	0.040*	
C29	0.0445 (4)	−0.4470 (2)	0.8487 (2)	0.0455 (8)	
H29A	−0.0148	−0.5135	0.8710	0.055*	
H29B	0.0071	−0.4024	0.8996	0.055*	
H29C	0.1559	−0.4676	0.8429	0.055*	
C30	−0.1511 (3)	−0.3555 (3)	0.7483 (2)	0.0472 (8)	
H30C	−0.1980	−0.3158	0.8023	0.057*	
H30B	−0.2034	−0.4244	0.7644	0.057*	
H30A	−0.1636	−0.3099	0.6812	0.057*	

### (I) trans-Dichloridotetrakis[1-(2,6-diisopropylphenyl)-1H-imidazole-κN3]iron(II) Atomic displacement parameters (Å^2^)

**Table:**

	*U*^11^	*U*^22^	*U*^33^	*U*^12^	*U*^13^	*U*^23^
Fe1	0.0218 (4)	0.0163 (3)	0.0257 (3)	−0.0025 (3)	−0.0081 (3)	−0.0043 (3)
Cl1	0.0265 (4)	0.0220 (4)	0.0379 (4)	0.0027 (3)	−0.0120 (3)	−0.0093 (3)
N1	0.0268 (14)	0.0244 (12)	0.0299 (12)	0.0021 (10)	−0.0099 (10)	−0.0066 (10)
N2	0.0325 (14)	0.0284 (12)	0.0217 (12)	0.0013 (11)	−0.0088 (10)	−0.0047 (10)
N3	0.0211 (13)	0.0185 (12)	0.0329 (12)	−0.0032 (9)	−0.0086 (10)	−0.0030 (10)
N4	0.0213 (13)	0.0189 (11)	0.0373 (13)	−0.0039 (10)	−0.0096 (10)	−0.0062 (10)
C1	0.0306 (17)	0.0260 (15)	0.0248 (15)	0.0020 (13)	−0.0061 (13)	−0.0080 (12)
C2	0.0389 (19)	0.0299 (16)	0.0362 (17)	0.0103 (14)	−0.0113 (14)	−0.0095 (14)
C3	0.045 (2)	0.0317 (16)	0.0340 (17)	0.0107 (15)	−0.0116 (14)	−0.0141 (13)
C4	0.0371 (18)	0.0250 (15)	0.0231 (15)	0.0061 (13)	−0.0082 (13)	−0.0069 (12)
C5	0.0391 (19)	0.0339 (16)	0.0271 (15)	0.0023 (14)	−0.0090 (14)	−0.0121 (13)
C6	0.045 (2)	0.050 (2)	0.0411 (18)	0.0104 (16)	−0.0220 (16)	−0.0140 (16)
C7	0.069 (3)	0.044 (2)	0.0357 (18)	0.0143 (18)	−0.0220 (18)	−0.0054 (16)
C8	0.064 (2)	0.0342 (18)	0.0362 (18)	−0.0012 (17)	−0.0106 (17)	0.0024 (14)
C9	0.0412 (19)	0.0334 (17)	0.0276 (15)	−0.0013 (14)	−0.0012 (14)	−0.0074 (13)
C10	0.045 (2)	0.0419 (18)	0.0443 (18)	−0.0084 (15)	−0.0189 (16)	−0.0069 (15)
C11A	0.076 (4)	0.051 (3)	0.041 (4)	−0.019 (3)	0.001 (3)	−0.006 (3)
C12A	0.112 (10)	0.050 (4)	0.073 (3)	−0.019 (5)	−0.008 (7)	−0.025 (3)
C11B	0.076 (4)	0.051 (3)	0.041 (4)	−0.019 (3)	0.001 (3)	−0.006 (3)
C12B	0.112 (10)	0.050 (4)	0.073 (3)	−0.019 (5)	−0.008 (7)	−0.025 (3)
C13	0.046 (2)	0.0368 (18)	0.0445 (19)	−0.0098 (15)	−0.0051 (16)	−0.0018 (15)
C14	0.060 (3)	0.074 (3)	0.089 (3)	−0.027 (2)	−0.007 (2)	−0.035 (2)
C15	0.059 (3)	0.064 (2)	0.064 (2)	−0.014 (2)	0.001 (2)	−0.0027 (19)
C16	0.0206 (16)	0.0251 (15)	0.0303 (16)	−0.0025 (12)	−0.0099 (13)	−0.0026 (12)
C17	0.0248 (16)	0.0184 (14)	0.0446 (17)	0.0005 (12)	−0.0095 (14)	−0.0032 (12)
C18	0.0214 (16)	0.0219 (15)	0.0510 (18)	0.0021 (12)	−0.0145 (14)	−0.0077 (13)
C19	0.0239 (16)	0.0168 (13)	0.0410 (16)	−0.0021 (12)	−0.0127 (13)	−0.0084 (12)
C20	0.0285 (17)	0.0274 (15)	0.0386 (16)	−0.0017 (12)	−0.0094 (13)	−0.0097 (13)
C21	0.041 (2)	0.0302 (17)	0.0468 (19)	−0.0017 (14)	−0.0055 (16)	−0.0195 (14)
C22	0.052 (2)	0.0234 (16)	0.059 (2)	−0.0089 (15)	−0.0063 (18)	−0.0134 (15)
C23	0.041 (2)	0.0232 (16)	0.0449 (18)	−0.0090 (14)	0.0001 (15)	−0.0074 (14)
C24	0.0247 (16)	0.0211 (14)	0.0395 (16)	−0.0043 (12)	−0.0061 (13)	−0.0042 (12)
C25	0.0353 (18)	0.0357 (17)	0.0360 (16)	−0.0063 (13)	−0.0062 (14)	−0.0127 (13)
C26	0.056 (2)	0.057 (2)	0.046 (2)	0.0057 (19)	−0.0088 (18)	−0.0019 (17)
C27	0.042 (2)	0.061 (2)	0.0399 (19)	0.0060 (17)	−0.0046 (16)	−0.0137 (17)
C28	0.0331 (18)	0.0251 (15)	0.0400 (17)	−0.0061 (13)	−0.0031 (14)	−0.0091 (13)
C29	0.050 (2)	0.0449 (19)	0.0418 (18)	−0.0058 (16)	−0.0098 (16)	−0.0114 (15)
C30	0.044 (2)	0.050 (2)	0.0468 (19)	0.0050 (16)	−0.0052 (16)	−0.0186 (16)

### (I) trans-Dichloridotetrakis[1-(2,6-diisopropylphenyl)-1H-imidazole-κN3]iron(II) Geometric parameters (Å, º)

**Table:**

Fe1—N3	2.161 (2)	C12B—H12D	0.9800
Fe1—N3^i^	2.161 (2)	C12B—H12E	0.9800
Fe1—N1	2.188 (2)	C12B—H12F	0.9800
Fe1—N1^i^	2.188 (2)	C13—C15	1.527 (4)
Fe1—Cl1	2.5356 (9)	C13—C14	1.532 (4)
Fe1—Cl1^i^	2.5356 (9)	C13—H13	1.0000
N1—C1	1.313 (3)	C14—H14A	0.9800
N1—C2	1.365 (3)	C14—H14B	0.9800
N2—C1	1.344 (3)	C14—H14C	0.9800
N2—C3	1.376 (3)	C15—H15A	0.9800
N2—C4	1.437 (3)	C15—H15B	0.9800
N3—C16	1.307 (3)	C15—H15C	0.9800
N3—C17	1.369 (3)	C16—H16	0.9500
N4—C16	1.342 (3)	C17—C18	1.362 (4)
N4—C18	1.371 (3)	C17—H17	0.9500
N4—C19	1.441 (3)	C18—H18	0.9500
C1—H1	0.9500	C19—C24	1.387 (4)
C2—C3	1.349 (4)	C19—C20	1.403 (4)
C2—H2	0.9500	C20—C21	1.385 (4)
C3—H3	0.9500	C20—C25	1.517 (4)
C4—C5	1.379 (4)	C21—C22	1.377 (4)
C4—C9	1.399 (4)	C21—H21	0.9500
C5—C6	1.400 (3)	C22—C23	1.367 (4)
C5—C10	1.517 (4)	C22—H22	0.9500
C6—C7	1.370 (4)	C23—C24	1.393 (4)
C6—H6	0.9500	C23—H23	0.9500
C7—C8	1.368 (4)	C24—C28	1.512 (4)
C7—H7	0.9500	C25—C26	1.508 (4)
C8—C9	1.390 (4)	C25—C27	1.513 (4)
C8—H8	0.9500	C25—H25	1.0000
C9—C13	1.505 (4)	C26—H26A	0.9800
C10—C12B	1.489 (17)	C26—H26B	0.9800
C10—C11A	1.509 (6)	C26—H26C	0.9800
C10—C12A	1.509 (8)	C27—H27C	0.9800
C10—C11B	1.553 (14)	C27—H27B	0.9800
C10—H10A	1.0000	C27—H27A	0.9800
C10—H10B	1.0000	C28—C30	1.515 (4)
C11A—H11A	0.9800	C28—C29	1.522 (4)
C11A—H11B	0.9800	C28—H28	1.0000
C11A—H11C	0.9800	C29—H29A	0.9800
C12A—H12A	0.9800	C29—H29B	0.9800
C12A—H12B	0.9800	C29—H29C	0.9800
C12A—H12C	0.9800	C30—H30C	0.9800
C11B—H11D	0.9800	C30—H30B	0.9800
C11B—H11E	0.9800	C30—H30A	0.9800
C11B—H11F	0.9800		
			
N3—Fe1—N3^i^	180.0	H12D—C12B—H12E	109.5
N3—Fe1—N1	85.67 (8)	C10—C12B—H12F	109.5
N3^i^—Fe1—N1	94.33 (8)	H12D—C12B—H12F	109.5
N3—Fe1—N1^i^	94.33 (8)	H12E—C12B—H12F	109.5
N3^i^—Fe1—N1^i^	85.67 (8)	C9—C13—C15	114.4 (3)
N1—Fe1—N1^i^	180.0	C9—C13—C14	111.1 (3)
N3—Fe1—Cl1	89.14 (6)	C15—C13—C14	108.7 (3)
N3^i^—Fe1—Cl1	90.86 (6)	C9—C13—H13	107.5
N1—Fe1—Cl1	89.63 (6)	C15—C13—H13	107.5
N1^i^—Fe1—Cl1	90.37 (6)	C14—C13—H13	107.5
N3—Fe1—Cl1^i^	90.86 (6)	C13—C14—H14A	109.5
N3^i^—Fe1—Cl1^i^	89.14 (6)	C13—C14—H14B	109.5
N1—Fe1—Cl1^i^	90.37 (6)	H14A—C14—H14B	109.5
N1^i^—Fe1—Cl1^i^	89.63 (6)	C13—C14—H14C	109.5
Cl1—Fe1—Cl1^i^	180.0	H14A—C14—H14C	109.5
C1—N1—C2	105.1 (2)	H14B—C14—H14C	109.5
C1—N1—Fe1	123.17 (17)	C13—C15—H15A	109.5
C2—N1—Fe1	131.53 (16)	C13—C15—H15B	109.5
C1—N2—C3	106.4 (2)	H15A—C15—H15B	109.5
C1—N2—C4	126.0 (2)	C13—C15—H15C	109.5
C3—N2—C4	127.5 (2)	H15A—C15—H15C	109.5
C16—N3—C17	105.2 (2)	H15B—C15—H15C	109.5
C16—N3—Fe1	127.93 (17)	N3—C16—N4	112.0 (2)
C17—N3—Fe1	125.16 (16)	N3—C16—H16	124.0
C16—N4—C18	107.2 (2)	N4—C16—H16	124.0
C16—N4—C19	126.7 (2)	C18—C17—N3	110.3 (2)
C18—N4—C19	126.1 (2)	C18—C17—H17	124.9
N1—C1—N2	112.1 (2)	N3—C17—H17	124.9
N1—C1—H1	124.0	C17—C18—N4	105.3 (2)
N2—C1—H1	124.0	C17—C18—H18	127.4
C3—C2—N1	110.5 (2)	N4—C18—H18	127.4
C3—C2—H2	124.8	C24—C19—C20	123.5 (2)
N1—C2—H2	124.8	C24—C19—N4	117.9 (2)
C2—C3—N2	105.9 (2)	C20—C19—N4	118.5 (2)
C2—C3—H3	127.0	C21—C20—C19	116.6 (2)
N2—C3—H3	127.0	C21—C20—C25	121.9 (3)
C5—C4—C9	123.6 (2)	C19—C20—C25	121.6 (2)
C5—C4—N2	118.8 (2)	C22—C21—C20	121.5 (3)
C9—C4—N2	117.5 (3)	C22—C21—H21	119.3
C4—C5—C6	117.3 (3)	C20—C21—H21	119.3
C4—C5—C10	122.4 (2)	C23—C22—C21	120.2 (3)
C6—C5—C10	120.3 (3)	C23—C22—H22	119.9
C7—C6—C5	120.2 (3)	C21—C22—H22	119.9
C7—C6—H6	119.9	C22—C23—C24	121.7 (3)
C5—C6—H6	119.9	C22—C23—H23	119.2
C8—C7—C6	121.2 (3)	C24—C23—H23	119.2
C8—C7—H7	119.4	C19—C24—C23	116.6 (3)
C6—C7—H7	119.4	C19—C24—C28	122.7 (2)
C7—C8—C9	121.1 (3)	C23—C24—C28	120.7 (2)
C7—C8—H8	119.4	C26—C25—C27	109.4 (2)
C9—C8—H8	119.4	C26—C25—C20	112.0 (2)
C8—C9—C4	116.5 (3)	C27—C25—C20	113.2 (2)
C8—C9—C13	122.0 (3)	C26—C25—H25	107.3
C4—C9—C13	121.5 (2)	C27—C25—H25	107.3
C12B—C10—C5	109.1 (14)	C20—C25—H25	107.3
C11A—C10—C5	111.8 (3)	C25—C26—H26A	109.5
C11A—C10—C12A	109.9 (4)	C25—C26—H26B	109.5
C5—C10—C12A	114.9 (5)	H26A—C26—H26B	109.5
C12B—C10—C11B	112.4 (13)	C25—C26—H26C	109.5
C5—C10—C11B	105.9 (7)	H26A—C26—H26C	109.5
C11A—C10—H10A	106.6	H26B—C26—H26C	109.5
C5—C10—H10A	106.6	C25—C27—H27C	109.5
C12A—C10—H10A	106.6	C25—C27—H27B	109.5
C12B—C10—H10B	109.8	H27C—C27—H27B	109.5
C5—C10—H10B	109.8	C25—C27—H27A	109.5
C11B—C10—H10B	109.8	H27C—C27—H27A	109.5
C10—C11A—H11A	109.5	H27B—C27—H27A	109.5
C10—C11A—H11B	109.5	C24—C28—C30	110.7 (2)
H11A—C11A—H11B	109.5	C24—C28—C29	112.6 (2)
C10—C11A—H11C	109.5	C30—C28—C29	110.6 (2)
H11A—C11A—H11C	109.5	C24—C28—H28	107.6
H11B—C11A—H11C	109.5	C30—C28—H28	107.6
C10—C12A—H12A	109.5	C29—C28—H28	107.6
C10—C12A—H12B	109.5	C28—C29—H29A	109.5
H12A—C12A—H12B	109.5	C28—C29—H29B	109.5
C10—C12A—H12C	109.5	H29A—C29—H29B	109.5
H12A—C12A—H12C	109.5	C28—C29—H29C	109.5
H12B—C12A—H12C	109.5	H29A—C29—H29C	109.5
C10—C11B—H11D	109.5	H29B—C29—H29C	109.5
C10—C11B—H11E	109.5	C28—C30—H30C	109.5
H11D—C11B—H11E	109.5	C28—C30—H30B	109.5
C10—C11B—H11F	109.5	H30C—C30—H30B	109.5
H11D—C11B—H11F	109.5	C28—C30—H30A	109.5
H11E—C11B—H11F	109.5	H30C—C30—H30A	109.5
C10—C12B—H12D	109.5	H30B—C30—H30A	109.5
C10—C12B—H12E	109.5		
			
C2—N1—C1—N2	−0.9 (3)	C8—C9—C13—C14	87.8 (3)
Fe1—N1—C1—N2	−176.06 (17)	C4—C9—C13—C14	−88.7 (3)
C3—N2—C1—N1	1.0 (3)	C17—N3—C16—N4	0.2 (3)
C4—N2—C1—N1	−176.7 (2)	Fe1—N3—C16—N4	−165.34 (16)
C1—N1—C2—C3	0.5 (3)	C18—N4—C16—N3	−0.3 (3)
Fe1—N1—C2—C3	175.1 (2)	C19—N4—C16—N3	178.4 (2)
N1—C2—C3—N2	0.1 (3)	C16—N3—C17—C18	0.0 (3)
C1—N2—C3—C2	−0.6 (3)	Fe1—N3—C17—C18	166.01 (18)
C4—N2—C3—C2	177.1 (3)	N3—C17—C18—N4	−0.1 (3)
C1—N2—C4—C5	−92.0 (3)	C16—N4—C18—C17	0.2 (3)
C3—N2—C4—C5	90.8 (3)	C19—N4—C18—C17	−178.5 (2)
C1—N2—C4—C9	86.0 (3)	C16—N4—C19—C24	101.2 (3)
C3—N2—C4—C9	−91.3 (3)	C18—N4—C19—C24	−80.3 (3)
C9—C4—C5—C6	1.9 (4)	C16—N4—C19—C20	−77.9 (3)
N2—C4—C5—C6	179.8 (2)	C18—N4—C19—C20	100.5 (3)
C9—C4—C5—C10	−175.9 (3)	C24—C19—C20—C21	2.3 (4)
N2—C4—C5—C10	1.9 (4)	N4—C19—C20—C21	−178.6 (2)
C4—C5—C6—C7	−0.2 (4)	C24—C19—C20—C25	−177.5 (2)
C10—C5—C6—C7	177.6 (3)	N4—C19—C20—C25	1.6 (4)
C5—C6—C7—C8	−1.5 (5)	C19—C20—C21—C22	−1.1 (4)
C6—C7—C8—C9	1.6 (5)	C25—C20—C21—C22	178.7 (3)
C7—C8—C9—C4	0.0 (4)	C20—C21—C22—C23	−0.6 (5)
C7—C8—C9—C13	−176.6 (3)	C21—C22—C23—C24	1.2 (5)
C5—C4—C9—C8	−1.8 (4)	C20—C19—C24—C23	−1.8 (4)
N2—C4—C9—C8	−179.7 (2)	N4—C19—C24—C23	179.2 (2)
C5—C4—C9—C13	174.8 (3)	C20—C19—C24—C28	178.5 (2)
N2—C4—C9—C13	−3.0 (4)	N4—C19—C24—C28	−0.6 (4)
C4—C5—C10—C12B	−112.5 (13)	C22—C23—C24—C19	0.0 (4)
C6—C5—C10—C12B	69.8 (14)	C22—C23—C24—C28	179.8 (3)
C4—C5—C10—C11A	96.3 (5)	C21—C20—C25—C26	102.9 (3)
C6—C5—C10—C11A	−81.4 (5)	C19—C20—C25—C26	−77.3 (3)
C4—C5—C10—C12A	−137.6 (5)	C21—C20—C25—C27	−21.3 (4)
C6—C5—C10—C12A	44.7 (6)	C19—C20—C25—C27	158.4 (3)
C4—C5—C10—C11B	126.4 (9)	C19—C24—C28—C30	102.5 (3)
C6—C5—C10—C11B	−51.4 (9)	C23—C24—C28—C30	−77.3 (3)
C8—C9—C13—C15	−35.7 (4)	C19—C24—C28—C29	−133.2 (3)
C4—C9—C13—C15	147.8 (3)	C23—C24—C28—C29	47.0 (4)

Symmetry code: (i) −*x*+1, −*y*, −*z*+1.

### (I) trans-Dichloridotetrakis[1-(2,6-diisopropylphenyl)-1H-imidazole-κN3]iron(II) Hydrogen-bond geometry (Å, º)

Cg3 and Cg4 are the centroids of rings C4–C9 and C19–C24, respectively.

**Table:**

*D*—H···*A*	*D*—H	H···*A*	*D*···*A*	*D*—H···*A*
C1—H1···Cl1^i^	0.95	2.62	3.257 (3)	125
C2—H2···Cl1	0.95	2.92	3.433 (3)	115
C16—H16···Cl1	0.95	2.76	3.294 (3)	117
C17—H17···Cl1^i^	0.95	2.82	3.375 (3)	118
C18—H18···Cl1^ii^	0.95	2.70	3.629 (3)	166
C27—H27*A*···*Cg*4^iii^	0.98	2.79	3.562 (4)	136
C30—H30*C*···*Cg*3^iv^	0.98	2.92	3.901 (4)	176

Symmetry codes: (i) −*x*+1, −*y*, −*z*+1; (ii) *x*−1, *y*, *z*; (iii) −*x*+1, −*y*−1, −*z*+1; (iv) −*x*, −*y*, −*z*+1.

### (II) trans-Dibromidotetrakis[1-(2,6-diisopropylphenyl)-1H-imidazole-κN3]iron(II) Crystal data

**Table:**

[FeBr_2_(C_15_H_20_N_2_)_4_]	*Z* = 1
*M**_r_* = 1128.99	*F*(000) = 592
Triclinic, *P*1	*D*_x_ = 1.279 Mg m^−^^3^
Hall symbol: -P 1	Mo *K*α radiation, λ = 0.71073 Å
*a* = 9.0391 (11) Å	Cell parameters from 7257 reflections
*b* = 12.7658 (11) Å	θ = 0.1–24.9°
*c* = 13.689 (2) Å	µ = 1.66 mm^−^^1^
α = 74.502 (9)°	*T* = 173 K
β = 74.481 (12)°	Plate, yellow
γ = 84.343 (9)°	0.20 × 0.17 × 0.10 mm
*V* = 1466.0 (3) Å^3^	

### (II) trans-Dibromidotetrakis[1-(2,6-diisopropylphenyl)-1H-imidazole-κN3]iron(II) Data collection

**Table:**

Stoe IPDS 2 diffractometer	5312 independent reflections
Radiation source: fine-focus sealed tube	3013 reflections with *I* > 2σ(*I*)
Plane graphite monochromator	*R*_int_ = 0.118
φ + ω scans	θ_max_ = 25.2°, θ_min_ = 1.6°
Absorption correction: multi-scan (MULscanABS in *PLATON*; Spek, 2009)	*h* = −10→10
*T*_min_ = 0.457, *T*_max_ = 0.496	*k* = −15→15
17613 measured reflections	*l* = −16→16

### (II) trans-Dibromidotetrakis[1-(2,6-diisopropylphenyl)-1H-imidazole-κN3]iron(II) Refinement

**Table:**

Refinement on *F*^2^	2 restraints
Least-squares matrix: full	Hydrogen site location: inferred from neighbouring sites
*R*[*F*^2^ > 2σ(*F*^2^)] = 0.046	H-atom parameters constrained
*wR*(*F*^2^) = 0.081	*w* = 1/[σ^2^(*F*_o_^2^) + (0.020*P*)^2^] where *P* = (*F*_o_^2^ + 2*F*_c_^2^)/3
*S* = 0.81	(Δ/σ)_max_ = 0.001
5312 reflections	Δρ_max_ = 0.59 e Å^−^^3^
350 parameters	Δρ_min_ = −0.64 e Å^−^^3^

### (II) trans-Dibromidotetrakis[1-(2,6-diisopropylphenyl)-1H-imidazole-κN3]iron(II) Special details

**Table:**

Geometry. All e.s.d.'s (except the e.s.d. in the dihedral angle between two l.s. planes) are estimated using the full covariance matrix. The cell e.s.d.'s are taken into account individually in the estimation of e.s.d.'s in distances, angles and torsion angles; correlations between e.s.d.'s in cell parameters are only used when they are defined by crystal symmetry. An approximate (isotropic) treatment of cell e.s.d.'s is used for estimating e.s.d.'s involving l.s. planes.

### (II) trans-Dibromidotetrakis[1-(2,6-diisopropylphenyl)-1H-imidazole-κN3]iron(II) Fractional atomic coordinates and isotropic or equivalent isotropic displacement parameters (Å^2^)

**Table:**

	*x*	*y*	*z*	*U*_iso_*/*U*_eq_	Occ. (<1)
Fe1	0.5000	0.0000	0.5000	0.0194 (2)	
Br1	0.70958 (6)	−0.16630 (4)	0.49630 (4)	0.02557 (14)	
N1	0.5093 (4)	0.0144 (3)	0.3354 (3)	0.0234 (8)	
N2	0.4486 (4)	0.0707 (3)	0.1838 (3)	0.0260 (9)	
N3	0.3197 (4)	−0.1141 (2)	0.5363 (3)	0.0203 (8)	
N4	0.2095 (4)	−0.2665 (3)	0.5529 (3)	0.0238 (8)	
C1	0.4300 (5)	0.0853 (3)	0.2808 (3)	0.0265 (11)	
H1	0.3663	0.1411	0.3060	0.032*	
C2	0.5844 (6)	−0.0508 (4)	0.2709 (4)	0.0356 (13)	
H2	0.6517	−0.1106	0.2890	0.043*	
C3	0.5470 (5)	−0.0162 (4)	0.1766 (4)	0.0362 (12)	
H3	0.5827	−0.0467	0.1180	0.043*	
C4	0.3836 (6)	0.1393 (4)	0.1024 (3)	0.0288 (11)	
C5	0.2450 (5)	0.1109 (3)	0.0933 (3)	0.0307 (11)	
C6	0.1847 (6)	0.1782 (4)	0.0137 (4)	0.0427 (13)	
H6	0.0900	0.1615	0.0046	0.051*	
C7	0.2617 (7)	0.2686 (4)	−0.0516 (4)	0.0457 (14)	
H7	0.2213	0.3127	−0.1068	0.055*	
C8	0.3959 (6)	0.2954 (4)	−0.0377 (4)	0.0448 (14)	
H8	0.4459	0.3592	−0.0828	0.054*	
C9	0.4613 (5)	0.2325 (4)	0.0401 (4)	0.0337 (12)	
C10	0.1572 (6)	0.0135 (4)	0.1677 (4)	0.0426 (13)	
H10A	0.2393	−0.0198	0.2040	0.051*	0.5
H1OB	0.2132	−0.0342	0.2179	0.051*	0.5
C11A	0.041 (2)	0.0434 (18)	0.2621 (10)	0.051 (4)	0.5
H11A	0.0954	0.0745	0.3004	0.061*	0.5
H11B	−0.0110	−0.0222	0.3087	0.061*	0.5
H11C	−0.0349	0.0967	0.2373	0.061*	0.5
C12A	0.153 (2)	−0.0733 (15)	0.1164 (19)	0.079 (6)	0.5
H12A	0.1077	−0.0442	0.0565	0.095*	0.5
H12B	0.0900	−0.1325	0.1664	0.095*	0.5
H12C	0.2573	−0.1012	0.0920	0.095*	0.5
C11B	0.001 (2)	0.0529 (19)	0.2171 (14)	0.100 (9)	0.5
H11D	0.0113	0.1088	0.2518	0.119*	0.5
H11E	−0.0553	−0.0081	0.2689	0.119*	0.5
H11F	−0.0544	0.0838	0.1631	0.119*	0.5
C12B	0.077 (2)	−0.0471 (16)	0.1146 (18)	0.059 (5)	0.5
H12D	0.0408	−0.1168	0.1629	0.070*	0.5
H12E	0.1487	−0.0603	0.0512	0.070*	0.5
H12F	−0.0115	−0.0030	0.0957	0.070*	0.5
C13	0.6051 (6)	0.2642 (4)	0.0597 (4)	0.0441 (13)	
H13	0.6536	0.1964	0.0959	0.053*	
C14	0.5652 (7)	0.3400 (5)	0.1332 (5)	0.077 (2)	
H14A	0.5271	0.4103	0.0971	0.093*	
H14B	0.6573	0.3508	0.1538	0.093*	
H14C	0.4857	0.3075	0.1957	0.093*	
C15	0.7247 (7)	0.3167 (5)	−0.0404 (5)	0.075 (2)	
H15A	0.7432	0.2709	−0.0898	0.090*	
H15B	0.8208	0.3241	−0.0229	0.090*	
H15C	0.6863	0.3887	−0.0723	0.090*	
C16	0.3330 (5)	−0.2203 (3)	0.5594 (3)	0.0246 (10)	
H16	0.4198	−0.2602	0.5785	0.030*	
C17	0.1796 (5)	−0.0909 (3)	0.5136 (4)	0.0300 (11)	
H17	0.1374	−0.0196	0.4938	0.036*	
C18	0.1090 (5)	−0.1837 (3)	0.5234 (3)	0.0312 (11)	
H18	0.0113	−0.1899	0.5123	0.037*	
C19	0.1856 (5)	−0.3798 (3)	0.5707 (3)	0.0255 (10)	
C20	0.2595 (5)	−0.4331 (3)	0.4920 (4)	0.0300 (11)	
C21	0.2295 (6)	−0.5416 (4)	0.5106 (4)	0.0414 (13)	
H21	0.2751	−0.5795	0.4583	0.050*	
C22	0.1343 (6)	−0.5962 (4)	0.6038 (4)	0.0485 (15)	
H22	0.1151	−0.6709	0.6152	0.058*	
C23	0.0676 (6)	−0.5425 (4)	0.6798 (4)	0.0440 (14)	
H23	0.0024	−0.5811	0.7435	0.053*	
C24	0.0923 (5)	−0.4344 (3)	0.6665 (4)	0.0302 (11)	
C25	0.3678 (6)	−0.3726 (4)	0.3904 (4)	0.0387 (12)	
H25	0.4311	−0.3249	0.4094	0.046*	
C26	0.2812 (7)	−0.2998 (5)	0.3166 (4)	0.0616 (18)	
H26A	0.2158	−0.3440	0.2976	0.074*	
H26B	0.3543	−0.2613	0.2533	0.074*	
H26C	0.2171	−0.2469	0.3510	0.074*	
C27	0.4778 (6)	−0.4493 (4)	0.3360 (4)	0.0540 (16)	
H27C	0.5567	−0.4072	0.2790	0.065*	
H27B	0.4210	−0.4902	0.3074	0.065*	
H27A	0.5268	−0.5000	0.3865	0.065*	
C28	0.0166 (5)	−0.3767 (3)	0.7519 (3)	0.0320 (11)	
H28	0.0661	−0.3050	0.7338	0.038*	
C29	0.0378 (6)	−0.4406 (4)	0.8581 (4)	0.0484 (14)	
H29A	−0.0214	−0.5070	0.8818	0.058*	
H29B	0.0017	−0.3961	0.9086	0.058*	
H29C	0.1469	−0.4599	0.8525	0.058*	
C30	−0.1524 (6)	−0.3552 (4)	0.7565 (4)	0.0467 (14)	
H30C	−0.1990	−0.3161	0.8104	0.056*	
H30B	−0.2033	−0.4245	0.7738	0.056*	
H30A	−0.1644	−0.3111	0.6884	0.056*	

### (II) trans-Dibromidotetrakis[1-(2,6-diisopropylphenyl)-1H-imidazole-κN3]iron(II) Atomic displacement parameters (Å^2^)

**Table:**

	*U*^11^	*U*^22^	*U*^33^	*U*^12^	*U*^13^	*U*^23^
Fe1	0.0196 (5)	0.0193 (5)	0.0200 (5)	−0.0042 (4)	−0.0058 (4)	−0.0044 (4)
Br1	0.0234 (3)	0.0243 (3)	0.0308 (3)	0.0020 (2)	−0.0083 (2)	−0.0093 (2)
N1	0.022 (2)	0.026 (2)	0.021 (2)	0.0000 (16)	−0.0046 (17)	−0.0062 (16)
N2	0.031 (2)	0.029 (2)	0.018 (2)	−0.0003 (17)	−0.0067 (17)	−0.0041 (16)
N3	0.018 (2)	0.0173 (19)	0.024 (2)	−0.0015 (15)	−0.0029 (16)	−0.0046 (15)
N4	0.021 (2)	0.022 (2)	0.027 (2)	−0.0030 (16)	−0.0038 (17)	−0.0052 (16)
C1	0.031 (3)	0.026 (3)	0.024 (3)	0.001 (2)	−0.007 (2)	−0.011 (2)
C2	0.037 (3)	0.038 (3)	0.030 (3)	0.007 (2)	−0.009 (2)	−0.008 (2)
C3	0.044 (3)	0.035 (3)	0.029 (3)	0.012 (2)	−0.008 (2)	−0.013 (2)
C4	0.038 (3)	0.029 (3)	0.020 (3)	0.005 (2)	−0.007 (2)	−0.010 (2)
C5	0.038 (3)	0.034 (3)	0.021 (3)	0.000 (2)	−0.010 (2)	−0.007 (2)
C6	0.042 (3)	0.059 (3)	0.033 (3)	0.007 (3)	−0.016 (3)	−0.018 (3)
C7	0.062 (4)	0.044 (3)	0.025 (3)	0.016 (3)	−0.014 (3)	−0.003 (2)
C8	0.055 (4)	0.045 (3)	0.025 (3)	0.002 (3)	−0.005 (3)	0.002 (2)
C9	0.035 (3)	0.036 (3)	0.025 (3)	0.003 (2)	−0.003 (2)	−0.006 (2)
C10	0.044 (3)	0.047 (3)	0.040 (3)	−0.011 (3)	−0.016 (3)	−0.008 (3)
C11A	0.076 (10)	0.050 (8)	0.016 (8)	−0.017 (7)	0.007 (7)	−0.007 (7)
C12A	0.093 (17)	0.046 (11)	0.081 (12)	−0.006 (11)	−0.002 (14)	−0.005 (9)
C11B	0.14 (2)	0.065 (11)	0.060 (15)	−0.045 (13)	0.042 (13)	−0.013 (13)
C12B	0.068 (13)	0.054 (12)	0.061 (9)	−0.006 (8)	−0.006 (10)	−0.035 (9)
C13	0.041 (3)	0.047 (3)	0.037 (3)	−0.011 (3)	−0.003 (3)	0.000 (2)
C14	0.064 (4)	0.088 (5)	0.088 (5)	−0.039 (4)	0.003 (4)	−0.045 (4)
C15	0.055 (4)	0.084 (5)	0.068 (5)	−0.020 (4)	0.006 (4)	−0.003 (4)
C16	0.017 (2)	0.031 (3)	0.025 (3)	−0.002 (2)	−0.007 (2)	−0.004 (2)
C17	0.021 (3)	0.020 (2)	0.043 (3)	−0.001 (2)	−0.005 (2)	−0.002 (2)
C18	0.028 (3)	0.023 (2)	0.039 (3)	−0.001 (2)	−0.005 (2)	−0.004 (2)
C19	0.023 (2)	0.018 (2)	0.037 (3)	−0.0024 (19)	−0.008 (2)	−0.007 (2)
C20	0.027 (3)	0.026 (3)	0.038 (3)	−0.004 (2)	−0.005 (2)	−0.011 (2)
C21	0.046 (3)	0.038 (3)	0.040 (3)	0.004 (3)	−0.003 (3)	−0.020 (3)
C22	0.055 (4)	0.027 (3)	0.058 (4)	−0.008 (3)	0.001 (3)	−0.016 (3)
C23	0.048 (3)	0.026 (3)	0.047 (3)	−0.014 (2)	0.011 (3)	−0.009 (2)
C24	0.030 (3)	0.018 (2)	0.040 (3)	−0.002 (2)	−0.002 (2)	−0.010 (2)
C25	0.039 (3)	0.045 (3)	0.034 (3)	−0.007 (2)	−0.004 (2)	−0.015 (2)
C26	0.057 (4)	0.069 (4)	0.040 (3)	0.002 (3)	0.003 (3)	0.001 (3)
C27	0.046 (4)	0.067 (4)	0.038 (3)	0.005 (3)	0.004 (3)	−0.011 (3)
C28	0.037 (3)	0.024 (2)	0.031 (3)	−0.004 (2)	−0.002 (2)	−0.004 (2)
C29	0.050 (4)	0.053 (3)	0.045 (3)	−0.002 (3)	−0.013 (3)	−0.016 (3)
C30	0.042 (3)	0.053 (3)	0.042 (3)	0.012 (3)	−0.008 (3)	−0.014 (3)

### (II) trans-Dibromidotetrakis[1-(2,6-diisopropylphenyl)-1H-imidazole-κN3]iron(II) Geometric parameters (Å, º)

**Table:**

Fe1—N3^i^	2.161 (3)	C12B—H12D	0.9800
Fe1—N3	2.161 (3)	C12B—H12E	0.9800
Fe1—N1	2.190 (3)	C12B—H12F	0.9800
Fe1—N1^i^	2.190 (3)	C13—C14	1.528 (7)
Fe1—Br1	2.7040 (5)	C13—C15	1.534 (7)
Fe1—Br1^i^	2.7040 (5)	C13—H13	1.0000
N1—C1	1.300 (5)	C14—H14A	0.9800
N1—C2	1.377 (5)	C14—H14B	0.9800
N2—C1	1.354 (5)	C14—H14C	0.9800
N2—C3	1.360 (5)	C15—H15A	0.9800
N2—C4	1.442 (5)	C15—H15B	0.9800
N3—C16	1.309 (5)	C15—H15C	0.9800
N3—C17	1.370 (5)	C16—H16	0.9500
N4—C16	1.346 (5)	C17—C18	1.359 (6)
N4—C18	1.373 (5)	C17—H17	0.9500
N4—C19	1.429 (5)	C18—H18	0.9500
C1—H1	0.9500	C19—C24	1.397 (6)
C2—C3	1.369 (6)	C19—C20	1.409 (6)
C2—H2	0.9500	C20—C21	1.381 (6)
C3—H3	0.9500	C20—C25	1.528 (6)
C4—C5	1.383 (6)	C21—C22	1.381 (7)
C4—C9	1.393 (6)	C21—H21	0.9500
C5—C6	1.396 (6)	C22—C23	1.371 (6)
C5—C10	1.515 (6)	C22—H22	0.9500
C6—C7	1.374 (7)	C23—C24	1.376 (6)
C6—H6	0.9500	C23—H23	0.9500
C7—C8	1.366 (7)	C24—C28	1.520 (6)
C7—H7	0.9500	C25—C26	1.509 (7)
C8—C9	1.384 (6)	C25—C27	1.517 (6)
C8—H8	0.9500	C25—H25	1.0000
C9—C13	1.508 (7)	C26—H26A	0.9800
C10—C12A	1.472 (16)	C26—H26B	0.9800
C10—C11B	1.492 (17)	C26—H26C	0.9800
C10—C12B	1.536 (18)	C27—H27C	0.9800
C10—C11A	1.541 (16)	C27—H27B	0.9800
C10—H10A	1.0000	C27—H27A	0.9800
C10—H1OB	1.0000	C28—C30	1.512 (6)
C11A—H11A	0.9800	C28—C29	1.517 (6)
C11A—H11B	0.9800	C28—H28	1.0000
C11A—H11C	0.9800	C29—H29A	0.9800
C12A—H12A	0.9800	C29—H29B	0.9800
C12A—H12B	0.9800	C29—H29C	0.9800
C12A—H12C	0.9800	C30—H30C	0.9800
C11B—H11D	0.9800	C30—H30B	0.9800
C11B—H11E	0.9800	C30—H30A	0.9800
C11B—H11F	0.9800		
			
N3^i^—Fe1—N3	180.0	H12D—C12B—H12E	109.5
N3^i^—Fe1—N1	93.99 (12)	C10—C12B—H12F	109.5
N3—Fe1—N1	86.01 (12)	H12D—C12B—H12F	109.5
N3^i^—Fe1—N1^i^	86.01 (12)	H12E—C12B—H12F	109.5
N3—Fe1—N1^i^	93.99 (12)	C9—C13—C14	110.4 (4)
N1—Fe1—N1^i^	180.0	C9—C13—C15	113.9 (5)
N3^i^—Fe1—Br1	90.60 (8)	C14—C13—C15	109.7 (5)
N3—Fe1—Br1	89.40 (8)	C9—C13—H13	107.5
N1—Fe1—Br1	89.70 (8)	C14—C13—H13	107.5
N1^i^—Fe1—Br1	90.30 (8)	C15—C13—H13	107.5
N3^i^—Fe1—Br1^i^	89.40 (8)	C13—C14—H14A	109.5
N3—Fe1—Br1^i^	90.60 (8)	C13—C14—H14B	109.5
N1—Fe1—Br1^i^	90.30 (8)	H14A—C14—H14B	109.5
N1^i^—Fe1—Br1^i^	89.70 (8)	C13—C14—H14C	109.5
Br1—Fe1—Br1^i^	180.0	H14A—C14—H14C	109.5
C1—N1—C2	105.3 (3)	H14B—C14—H14C	109.5
C1—N1—Fe1	124.7 (3)	C13—C15—H15A	109.5
C2—N1—Fe1	129.8 (3)	C13—C15—H15B	109.5
C1—N2—C3	106.8 (4)	H15A—C15—H15B	109.5
C1—N2—C4	125.9 (3)	C13—C15—H15C	109.5
C3—N2—C4	127.2 (4)	H15A—C15—H15C	109.5
C16—N3—C17	104.8 (3)	H15B—C15—H15C	109.5
C16—N3—Fe1	127.3 (3)	N3—C16—N4	112.2 (4)
C17—N3—Fe1	126.0 (3)	N3—C16—H16	123.9
C16—N4—C18	107.1 (3)	N4—C16—H16	123.9
C16—N4—C19	127.6 (3)	C18—C17—N3	110.8 (4)
C18—N4—C19	125.3 (4)	C18—C17—H17	124.6
N1—C1—N2	112.4 (4)	N3—C17—H17	124.6
N1—C1—H1	123.8	C17—C18—N4	105.1 (4)
N2—C1—H1	123.8	C17—C18—H18	127.5
C3—C2—N1	109.5 (4)	N4—C18—H18	127.5
C3—C2—H2	125.3	C24—C19—C20	122.3 (4)
N1—C2—H2	125.3	C24—C19—N4	118.7 (4)
N2—C3—C2	106.0 (4)	C20—C19—N4	118.9 (4)
N2—C3—H3	127.0	C21—C20—C19	117.2 (4)
C2—C3—H3	127.0	C21—C20—C25	121.7 (4)
C5—C4—C9	123.8 (4)	C19—C20—C25	121.1 (4)
C5—C4—N2	118.2 (4)	C20—C21—C22	121.2 (4)
C9—C4—N2	118.0 (4)	C20—C21—H21	119.4
C4—C5—C6	117.1 (4)	C22—C21—H21	119.4
C4—C5—C10	122.5 (4)	C23—C22—C21	120.0 (5)
C6—C5—C10	120.3 (5)	C23—C22—H22	120.0
C7—C6—C5	120.4 (5)	C21—C22—H22	120.0
C7—C6—H6	119.8	C22—C23—C24	121.9 (5)
C5—C6—H6	119.8	C22—C23—H23	119.1
C8—C7—C6	120.5 (5)	C24—C23—H23	119.1
C8—C7—H7	119.7	C23—C24—C19	117.3 (4)
C6—C7—H7	119.7	C23—C24—C28	120.8 (4)
C7—C8—C9	122.0 (5)	C19—C24—C28	121.9 (4)
C7—C8—H8	119.0	C26—C25—C27	110.5 (4)
C9—C8—H8	119.0	C26—C25—C20	111.9 (4)
C8—C9—C4	116.1 (5)	C27—C25—C20	112.3 (4)
C8—C9—C13	122.5 (5)	C26—C25—H25	107.3
C4—C9—C13	121.4 (4)	C27—C25—H25	107.3
C12A—C10—C5	112.6 (10)	C20—C25—H25	107.3
C11B—C10—C5	108.1 (10)	C25—C26—H26A	109.5
C11B—C10—C12B	86.9 (11)	C25—C26—H26B	109.5
C5—C10—C12B	113.7 (10)	H26A—C26—H26B	109.5
C12A—C10—C11A	129.6 (13)	C25—C26—H26C	109.5
C5—C10—C11A	112.4 (9)	H26A—C26—H26C	109.5
C12A—C10—H10A	97.7	H26B—C26—H26C	109.5
C5—C10—H10A	97.7	C25—C27—H27C	109.5
C11A—C10—H10A	97.7	C25—C27—H27B	109.5
C11B—C10—H1OB	115.0	H27C—C27—H27B	109.5
C5—C10—H1OB	115.0	C25—C27—H27A	109.5
C12B—C10—H1OB	115.0	H27C—C27—H27A	109.5
C10—C11A—H11A	109.5	H27B—C27—H27A	109.5
C10—C11A—H11B	109.5	C30—C28—C29	110.3 (4)
H11A—C11A—H11B	109.5	C30—C28—C24	110.4 (4)
C10—C11A—H11C	109.5	C29—C28—C24	112.0 (4)
H11A—C11A—H11C	109.5	C30—C28—H28	108.0
H11B—C11A—H11C	109.5	C29—C28—H28	108.0
C10—C12A—H12A	109.5	C24—C28—H28	108.0
C10—C12A—H12B	109.5	C28—C29—H29A	109.5
H12A—C12A—H12B	109.5	C28—C29—H29B	109.5
C10—C12A—H12C	109.5	H29A—C29—H29B	109.5
H12A—C12A—H12C	109.5	C28—C29—H29C	109.5
H12B—C12A—H12C	109.5	H29A—C29—H29C	109.5
C10—C11B—H11D	109.5	H29B—C29—H29C	109.5
C10—C11B—H11E	109.5	C28—C30—H30C	109.5
H11D—C11B—H11E	109.5	C28—C30—H30B	109.5
C10—C11B—H11F	109.5	H30C—C30—H30B	109.5
H11D—C11B—H11F	109.5	C28—C30—H30A	109.5
H11E—C11B—H11F	109.5	H30C—C30—H30A	109.5
C10—C12B—H12D	109.5	H30B—C30—H30A	109.5
C10—C12B—H12E	109.5		
			
C2—N1—C1—N2	−0.1 (5)	C8—C9—C13—C15	−38.0 (7)
Fe1—N1—C1—N2	−175.1 (3)	C4—C9—C13—C15	144.3 (5)
C3—N2—C1—N1	0.1 (5)	C17—N3—C16—N4	−0.1 (5)
C4—N2—C1—N1	−176.3 (4)	Fe1—N3—C16—N4	−164.9 (3)
C1—N1—C2—C3	0.0 (5)	C18—N4—C16—N3	0.0 (5)
Fe1—N1—C2—C3	174.7 (3)	C19—N4—C16—N3	178.8 (4)
C1—N2—C3—C2	−0.1 (5)	C16—N3—C17—C18	0.1 (5)
C4—N2—C3—C2	176.2 (4)	Fe1—N3—C17—C18	165.2 (3)
N1—C2—C3—N2	0.0 (6)	N3—C17—C18—N4	−0.1 (5)
C1—N2—C4—C5	−94.1 (5)	C16—N4—C18—C17	0.1 (4)
C3—N2—C4—C5	90.3 (6)	C19—N4—C18—C17	−178.7 (4)
C1—N2—C4—C9	83.6 (6)	C16—N4—C19—C24	100.9 (5)
C3—N2—C4—C9	−92.0 (5)	C18—N4—C19—C24	−80.5 (5)
C9—C4—C5—C6	2.8 (7)	C16—N4—C19—C20	−77.7 (6)
N2—C4—C5—C6	−179.7 (4)	C18—N4—C19—C20	100.9 (5)
C9—C4—C5—C10	−175.2 (4)	C24—C19—C20—C21	3.6 (7)
N2—C4—C5—C10	2.3 (6)	N4—C19—C20—C21	−177.9 (4)
C4—C5—C6—C7	−0.2 (6)	C24—C19—C20—C25	−176.6 (4)
C10—C5—C6—C7	177.8 (4)	N4—C19—C20—C25	1.9 (6)
C5—C6—C7—C8	−1.8 (7)	C19—C20—C21—C22	−1.8 (7)
C6—C7—C8—C9	1.4 (8)	C25—C20—C21—C22	178.4 (5)
C7—C8—C9—C4	1.0 (7)	C20—C21—C22—C23	0.1 (8)
C7—C8—C9—C13	−176.9 (5)	C21—C22—C23—C24	0.0 (8)
C5—C4—C9—C8	−3.1 (7)	C22—C23—C24—C19	1.7 (8)
N2—C4—C9—C8	179.4 (4)	C22—C23—C24—C28	179.8 (5)
C5—C4—C9—C13	174.7 (4)	C20—C19—C24—C23	−3.5 (7)
N2—C4—C9—C13	−2.8 (6)	N4—C19—C24—C23	177.9 (4)
C4—C5—C10—C12A	−112.3 (9)	C20—C19—C24—C28	178.3 (4)
C6—C5—C10—C12A	69.8 (9)	N4—C19—C24—C28	−0.2 (6)
C4—C5—C10—C11B	122.2 (9)	C21—C20—C25—C26	103.5 (5)
C6—C5—C10—C11B	−55.7 (10)	C19—C20—C25—C26	−76.2 (6)
C4—C5—C10—C12B	−143.2 (7)	C21—C20—C25—C27	−21.4 (7)
C6—C5—C10—C12B	39.0 (8)	C19—C20—C25—C27	158.8 (4)
C4—C5—C10—C11A	91.2 (8)	C23—C24—C28—C30	−74.1 (6)
C6—C5—C10—C11A	−86.7 (8)	C19—C24—C28—C30	104.0 (5)
C8—C9—C13—C14	86.1 (6)	C23—C24—C28—C29	49.2 (6)
C4—C9—C13—C14	−91.6 (6)	C19—C24—C28—C29	−132.7 (5)

Symmetry code: (i) −*x*+1, −*y*, −*z*+1.

### (II) trans-Dibromidotetrakis[1-(2,6-diisopropylphenyl)-1H-imidazole-κN3]iron(II) Hydrogen-bond geometry (Å, º)

Cg3 and Cg4 are the centroids of rings C4–C9 and C19–C24, respectively.

**Table:**

*D*—H···*A*	*D*—H	H···*A*	*D*···*A*	*D*—H···*A*
C1—H1···Br1^i^	0.95	2.71	3.368 (4)	127
C2—H2···Br1	0.95	2.91	3.477 (5)	119
C16—H16···Br1	0.95	2.81	3.373 (4)	119
C17—H17···Br1^i^	0.95	2.91	3.484 (4)	120
C18—H18···Br1^ii^	0.95	2.77	3.707 (5)	167
C27—H27*A*···*Cg*4^iii^	0.98	2.92	3.639 (6)	131
C30—H30*C*···*Cg*3^iv^	0.98	2.88	3.862 (6)	177

Symmetry codes: (i) −*x*+1, −*y*, −*z*+1; (ii) *x*−1, *y*, *z*; (iii) −*x*+1, −*y*−1, −*z*+1; (iv) −*x*, −*y*, −*z*+1.

### (IIb) trans-Dibromidotetrakis[1-(2,6-diisopropylphenyl)-1H-imidazole-κN3]iron(II) diethyl ether disolvate Crystal data

**Table:**

[FeBr_2_(C_15_H_20_N_2_)_4_]·2C_4_H_10_O	*Z* = 1
*M**_r_* = 1277.22	*F*(000) = 676
Triclinic, *P*1	*D*_x_ = 1.204 Mg m^−^^3^
Hall symbol: -P 1	Mo *K*α radiation, λ = 0.71073 Å
*a* = 11.6710 (8) Å	Cell parameters from 25508 reflections
*b* = 12.4758 (9) Å	θ = 0.1–24.9°
*c* = 13.5759 (10) Å	µ = 1.39 mm^−^^1^
α = 64.464 (5)°	*T* = 173 K
β = 81.515 (6)°	Block, yellow
γ = 88.982 (6)°	0.50 × 0.50 × 0.50 mm
*V* = 1761.8 (2) Å^3^	

### (IIb) trans-Dibromidotetrakis[1-(2,6-diisopropylphenyl)-1H-imidazole-κN3]iron(II) diethyl ether disolvate Data collection

**Table:**

Stoe IPDS 2 diffractometer	6374 independent reflections
Radiation source: fine-focus sealed tube	5714 reflections with *I* > 2σ(*I*)
Plane graphite monochromator	*R*_int_ = 0.030
φ + ω scans	θ_max_ = 25.3°, θ_min_ = 1.7°
Absorption correction: multi-scan (MULscanABS in *PLATON*; Spek, 2009)	*h* = −14→14
*T*_min_ = 0.557, *T*_max_ = 0.672	*k* = −14→14
15799 measured reflections	*l* = −15→16

### (IIb) trans-Dibromidotetrakis[1-(2,6-diisopropylphenyl)-1H-imidazole-κN3]iron(II) diethyl ether disolvate Refinement

**Table:**

Refinement on *F*^2^	0 restraints
Least-squares matrix: full	Hydrogen site location: inferred from neighbouring sites
*R*[*F*^2^ > 2σ(*F*^2^)] = 0.031	H-atom parameters constrained
*wR*(*F*^2^) = 0.077	*w* = 1/[σ^2^(*F*_o_^2^) + (0.0431*P*)^2^ + 0.7229*P*] where *P* = (*F*_o_^2^ + 2*F*_c_^2^)/3
*S* = 1.03	(Δ/σ)_max_ = 0.001
6374 reflections	Δρ_max_ = 0.44 e Å^−^^3^
378 parameters	Δρ_min_ = −0.37 e Å^−^^3^

### (IIb) trans-Dibromidotetrakis[1-(2,6-diisopropylphenyl)-1H-imidazole-κN3]iron(II) diethyl ether disolvate Special details

**Table:**

Geometry. All e.s.d.'s (except the e.s.d. in the dihedral angle between two l.s. planes) are estimated using the full covariance matrix. The cell e.s.d.'s are taken into account individually in the estimation of e.s.d.'s in distances, angles and torsion angles; correlations between e.s.d.'s in cell parameters are only used when they are defined by crystal symmetry. An approximate (isotropic) treatment of cell e.s.d.'s is used for estimating e.s.d.'s involving l.s. planes.

### (IIb) trans-Dibromidotetrakis[1-(2,6-diisopropylphenyl)-1H-imidazole-κN3]iron(II) diethyl ether disolvate Fractional atomic coordinates and isotropic or equivalent isotropic displacement parameters (Å^2^)

**Table:**

	*x*	*y*	*z*	*U*_iso_*/*U*_eq_	Occ. (<1)
Fe1	0.5000	1.0000	0.5000	0.02343 (9)	
Br1	0.37430 (2)	0.79434 (2)	0.64396 (2)	0.02939 (7)	
N1	0.46601 (13)	1.07022 (14)	0.62346 (13)	0.0270 (3)	
N2	0.48075 (14)	1.18785 (14)	0.70489 (13)	0.0278 (3)	
N3	0.65641 (13)	0.93155 (14)	0.57112 (13)	0.0271 (3)	
N4	0.75321 (13)	0.80847 (14)	0.69892 (13)	0.0277 (3)	
C1	0.50151 (17)	1.17670 (17)	0.60955 (16)	0.0285 (4)	
H1	0.5371	1.2375	0.5410	0.034*	
C2	0.42078 (17)	1.01038 (18)	0.73346 (17)	0.0315 (4)	
H2	0.3885	0.9310	0.7684	0.038*	
C3	0.42911 (18)	1.08153 (18)	0.78454 (17)	0.0330 (4)	
H3	0.4042	1.0619	0.8604	0.040*	
C4	0.51202 (18)	1.29018 (17)	0.72041 (16)	0.0293 (4)	
C5	0.61769 (19)	1.29155 (18)	0.75702 (17)	0.0344 (4)	
C6	0.6449 (2)	1.3901 (2)	0.77407 (19)	0.0437 (5)	
H6	0.7154	1.3940	0.7998	0.052*	
C7	0.5708 (3)	1.4816 (2)	0.75409 (19)	0.0491 (6)	
H7	0.5910	1.5477	0.7664	0.059*	
C8	0.4679 (2)	1.47935 (19)	0.71658 (19)	0.0462 (6)	
H8	0.4186	1.5440	0.7027	0.055*	
C9	0.43549 (19)	1.38246 (18)	0.69876 (17)	0.0355 (5)	
C10	0.7014 (2)	1.1920 (2)	0.7763 (2)	0.0439 (5)	
H10	0.6593	1.1250	0.7725	0.053*	
C11	0.8047 (3)	1.2317 (3)	0.6846 (3)	0.0823 (11)	
H11A	0.8487	1.2969	0.6861	0.099*	
H11B	0.7776	1.2589	0.6131	0.099*	
H11C	0.8547	1.1648	0.6952	0.099*	
C12	0.7368 (4)	1.1444 (4)	0.8904 (3)	0.0943 (13)	
H12C	0.7884	1.0789	0.9003	0.113*	
H12B	0.6674	1.1154	0.9467	0.113*	
H12A	0.7773	1.2081	0.8976	0.113*	
C13	0.3209 (2)	1.3778 (2)	0.6609 (2)	0.0454 (5)	
H13	0.3215	1.3104	0.6396	0.054*	
C14	0.2197 (3)	1.3530 (3)	0.7540 (3)	0.0751 (10)	
H14A	0.2156	1.4191	0.7751	0.090*	
H14B	0.2313	1.2792	0.8180	0.090*	
H14C	0.1472	1.3448	0.7290	0.090*	
C15	0.3026 (3)	1.4921 (3)	0.5592 (2)	0.0691 (8)	
H15A	0.3672	1.5066	0.4992	0.083*	
H15B	0.2992	1.5595	0.5784	0.083*	
H15C	0.2297	1.4831	0.5351	0.083*	
C16	0.66540 (16)	0.82018 (17)	0.64133 (16)	0.0271 (4)	
H16	0.6160	0.7559	0.6503	0.033*	
C17	0.74340 (17)	0.99506 (18)	0.58507 (18)	0.0339 (4)	
H17	0.7588	1.0786	0.5454	0.041*	
C18	0.80359 (17)	0.92076 (18)	0.66367 (18)	0.0347 (5)	
H18	0.8673	0.9417	0.6892	0.042*	
C19	0.77470 (17)	0.70242 (18)	0.79383 (18)	0.0336 (4)	
C20	0.7010 (2)	0.67551 (19)	0.89322 (19)	0.0395 (5)	
C21	0.7219 (3)	0.5728 (2)	0.9846 (2)	0.0537 (6)	
H21	0.6734	0.5507	1.0537	0.064*	
C22	0.8125 (3)	0.5027 (2)	0.9759 (3)	0.0635 (8)	
H22	0.8257	0.4336	1.0392	0.076*	
C23	0.8837 (2)	0.5320 (2)	0.8765 (3)	0.0563 (7)	
H23	0.9452	0.4826	0.8722	0.068*	
C24	0.86670 (18)	0.6333 (2)	0.7823 (2)	0.0417 (5)	
C25	0.6018 (2)	0.7531 (2)	0.90310 (19)	0.0436 (5)	
H25	0.6034	0.8222	0.8292	0.052*	
C26	0.6170 (3)	0.8022 (3)	0.9863 (2)	0.0613 (7)	
H26A	0.6150	0.7362	1.0598	0.074*	
H26B	0.5540	0.8546	0.9883	0.074*	
H26C	0.6916	0.8474	0.9640	0.074*	
C27	0.4846 (2)	0.6854 (2)	0.9333 (2)	0.0513 (6)	
H27C	0.4228	0.7357	0.9430	0.062*	
H27B	0.4834	0.6129	1.0023	0.062*	
H27A	0.4724	0.6639	0.8740	0.062*	
C28	0.9445 (2)	0.6657 (2)	0.6726 (2)	0.0525 (7)	
H28	0.9153	0.7391	0.6165	0.063*	
C29	0.9413 (3)	0.5689 (3)	0.6332 (3)	0.0709 (8)	
H29A	0.9677	0.4951	0.6878	0.085*	
H29B	0.9924	0.5936	0.5623	0.085*	
H29C	0.8618	0.5555	0.6243	0.085*	
C30	1.0691 (3)	0.6943 (4)	0.6787 (4)	0.0895 (12)	
H30C	1.1168	0.7180	0.6062	0.107*	
H30B	1.1001	0.6237	0.7334	0.107*	
H30A	1.0706	0.7595	0.7006	0.107*	
O1	0.96877 (19)	1.0413 (2)	0.2300 (2)	0.0818 (7)	
C31	0.9192 (4)	1.1111 (4)	0.1369 (4)	0.1043 (14)	
H31A	0.8628	1.1632	0.1541	0.125*	
H31B	0.8777	1.0599	0.1133	0.125*	
C32	1.0118 (4)	1.1834 (5)	0.0486 (4)	0.1199 (18)	
H32A	1.0544	1.2315	0.0737	0.180*	
H32B	0.9780	1.2359	−0.0165	0.180*	
H32C	1.0650	1.1313	0.0294	0.180*	
C33A	0.8915 (7)	0.9778 (10)	0.3432 (8)	0.0846 (13)	0.5
H33A	0.9359	0.9672	0.4033	0.102*	0.5
H33B	0.8238	1.0252	0.3480	0.102*	0.5
C33B	0.8679 (7)	0.9729 (10)	0.2985 (8)	0.0846 (13)	0.5
H33D	0.8287	0.9376	0.2587	0.102*	0.5
H33E	0.8132	1.0241	0.3190	0.102*	0.5
C34A	0.8529 (6)	0.8612 (8)	0.3533 (7)	0.0846 (13)	0.5
H34A	0.8077	0.8729	0.2943	0.127*	0.5
H34B	0.8045	0.8173	0.4252	0.127*	0.5
H34C	0.9207	0.8158	0.3469	0.127*	0.5
C34B	0.9022 (6)	0.8809 (8)	0.3956 (7)	0.0846 (13)	0.5
H34D	0.9424	0.8211	0.3766	0.127*	0.5
H34E	0.8332	0.8432	0.4507	0.127*	0.5
H34F	0.9543	0.9152	0.4260	0.127*	0.5

### (IIb) trans-Dibromidotetrakis[1-(2,6-diisopropylphenyl)-1H-imidazole-κN3]iron(II) diethyl ether disolvate Atomic displacement parameters (Å^2^)

**Table:**

	*U*^11^	*U*^22^	*U*^33^	*U*^12^	*U*^13^	*U*^23^
Fe1	0.02406 (18)	0.01970 (18)	0.0303 (2)	0.00385 (13)	−0.00785 (14)	−0.01329 (16)
Br1	0.02990 (11)	0.02252 (10)	0.03696 (12)	0.00061 (7)	−0.00611 (7)	−0.01369 (8)
N1	0.0280 (8)	0.0245 (8)	0.0330 (9)	0.0040 (6)	−0.0080 (6)	−0.0156 (7)
N2	0.0342 (8)	0.0222 (8)	0.0294 (8)	0.0015 (6)	−0.0043 (7)	−0.0135 (7)
N3	0.0247 (8)	0.0249 (8)	0.0330 (9)	0.0024 (6)	−0.0068 (6)	−0.0132 (7)
N4	0.0250 (8)	0.0253 (8)	0.0329 (9)	0.0037 (6)	−0.0086 (6)	−0.0115 (7)
C1	0.0331 (10)	0.0238 (9)	0.0314 (10)	0.0026 (7)	−0.0039 (8)	−0.0149 (8)
C2	0.0347 (10)	0.0243 (10)	0.0351 (11)	−0.0027 (8)	−0.0043 (8)	−0.0125 (8)
C3	0.0412 (11)	0.0268 (10)	0.0301 (10)	−0.0038 (8)	−0.0016 (8)	−0.0125 (9)
C4	0.0423 (11)	0.0216 (9)	0.0262 (10)	−0.0019 (8)	−0.0006 (8)	−0.0135 (8)
C5	0.0438 (11)	0.0286 (10)	0.0300 (10)	−0.0056 (9)	−0.0018 (9)	−0.0128 (9)
C6	0.0598 (14)	0.0357 (12)	0.0372 (12)	−0.0131 (10)	−0.0058 (10)	−0.0173 (10)
C7	0.0852 (19)	0.0279 (11)	0.0364 (12)	−0.0125 (11)	−0.0013 (12)	−0.0179 (10)
C8	0.0772 (17)	0.0226 (10)	0.0358 (12)	0.0077 (10)	0.0014 (11)	−0.0133 (9)
C9	0.0493 (12)	0.0251 (10)	0.0288 (10)	0.0055 (9)	0.0004 (9)	−0.0109 (9)
C10	0.0427 (12)	0.0370 (12)	0.0562 (14)	0.0005 (10)	−0.0146 (11)	−0.0219 (11)
C11	0.0598 (18)	0.062 (2)	0.117 (3)	0.0044 (15)	0.0155 (18)	−0.040 (2)
C12	0.121 (3)	0.090 (3)	0.086 (3)	0.050 (2)	−0.059 (2)	−0.039 (2)
C13	0.0495 (13)	0.0399 (13)	0.0452 (13)	0.0150 (10)	−0.0078 (10)	−0.0173 (11)
C14	0.0509 (16)	0.098 (3)	0.0563 (17)	0.0181 (16)	−0.0052 (13)	−0.0164 (17)
C15	0.082 (2)	0.068 (2)	0.0461 (16)	0.0163 (16)	−0.0181 (14)	−0.0116 (14)
C16	0.0258 (9)	0.0245 (9)	0.0340 (10)	0.0034 (7)	−0.0076 (7)	−0.0145 (8)
C17	0.0312 (10)	0.0255 (10)	0.0435 (12)	−0.0023 (8)	−0.0101 (9)	−0.0119 (9)
C18	0.0289 (10)	0.0306 (11)	0.0454 (12)	−0.0002 (8)	−0.0137 (9)	−0.0146 (9)
C19	0.0340 (10)	0.0260 (10)	0.0394 (11)	0.0025 (8)	−0.0165 (9)	−0.0095 (9)
C20	0.0491 (13)	0.0320 (11)	0.0372 (12)	0.0002 (9)	−0.0137 (10)	−0.0124 (10)
C21	0.0744 (18)	0.0425 (14)	0.0386 (13)	0.0002 (12)	−0.0210 (12)	−0.0084 (11)
C22	0.081 (2)	0.0366 (14)	0.0631 (18)	0.0086 (13)	−0.0413 (16)	−0.0030 (13)
C23	0.0499 (14)	0.0365 (13)	0.078 (2)	0.0128 (11)	−0.0308 (14)	−0.0146 (13)
C24	0.0311 (11)	0.0319 (11)	0.0608 (15)	0.0057 (9)	−0.0182 (10)	−0.0155 (11)
C25	0.0570 (14)	0.0409 (13)	0.0316 (11)	0.0053 (10)	−0.0056 (10)	−0.0148 (10)
C26	0.0748 (19)	0.0630 (18)	0.0591 (17)	0.0011 (14)	−0.0097 (14)	−0.0386 (15)
C27	0.0556 (15)	0.0568 (16)	0.0425 (13)	0.0037 (12)	−0.0070 (11)	−0.0226 (12)
C28	0.0308 (11)	0.0390 (13)	0.0796 (19)	0.0078 (9)	−0.0025 (11)	−0.0203 (13)
C29	0.0612 (18)	0.069 (2)	0.086 (2)	−0.0062 (15)	−0.0008 (16)	−0.0394 (18)
C30	0.0403 (15)	0.092 (3)	0.152 (4)	−0.0108 (15)	0.0059 (18)	−0.073 (3)
O1	0.0574 (12)	0.0860 (17)	0.0837 (16)	0.0012 (11)	−0.0191 (11)	−0.0169 (13)
C31	0.083 (3)	0.112 (3)	0.122 (4)	0.000 (2)	−0.048 (3)	−0.045 (3)
C32	0.107 (3)	0.132 (4)	0.089 (3)	−0.028 (3)	−0.033 (3)	−0.012 (3)
C33A	0.053 (2)	0.094 (3)	0.100 (4)	0.003 (2)	0.001 (2)	−0.040 (3)
C33B	0.053 (2)	0.094 (3)	0.100 (4)	0.003 (2)	0.001 (2)	−0.040 (3)
C34A	0.053 (2)	0.094 (3)	0.100 (4)	0.003 (2)	0.001 (2)	−0.040 (3)
C34B	0.053 (2)	0.094 (3)	0.100 (4)	0.003 (2)	0.001 (2)	−0.040 (3)

### (IIb) trans-Dibromidotetrakis[1-(2,6-diisopropylphenyl)-1H-imidazole-κN3]iron(II) diethyl ether disolvate Geometric parameters (Å, º)

**Table:**

Fe1—N3^i^	2.1789 (15)	C18—H18	0.9500
Fe1—N3	2.1789 (15)	C19—C20	1.397 (3)
Fe1—N1	2.1889 (16)	C19—C24	1.399 (3)
Fe1—N1^i^	2.1889 (16)	C20—C21	1.396 (3)
Fe1—Br1	2.7422 (3)	C20—C25	1.523 (3)
Fe1—Br1^i^	2.7422 (3)	C21—C22	1.384 (4)
N1—C1	1.324 (2)	C21—H21	0.9500
N1—C2	1.373 (3)	C22—C23	1.378 (4)
N2—C1	1.347 (2)	C22—H22	0.9500
N2—C3	1.375 (3)	C23—C24	1.395 (3)
N2—C4	1.442 (2)	C23—H23	0.9500
N3—C16	1.316 (2)	C24—C28	1.516 (4)
N3—C17	1.382 (2)	C25—C27	1.527 (4)
N4—C16	1.347 (2)	C25—C26	1.530 (3)
N4—C18	1.380 (3)	C25—H25	1.0000
N4—C19	1.445 (3)	C26—H26A	0.9800
C1—H1	0.9500	C26—H26B	0.9800
C2—C3	1.354 (3)	C26—H26C	0.9800
C2—H2	0.9500	C27—H27C	0.9800
C3—H3	0.9500	C27—H27B	0.9800
C4—C5	1.399 (3)	C27—H27A	0.9800
C4—C9	1.400 (3)	C28—C29	1.521 (4)
C5—C6	1.395 (3)	C28—C30	1.526 (4)
C5—C10	1.522 (3)	C28—H28	1.0000
C6—C7	1.376 (4)	C29—H29A	0.9800
C6—H6	0.9500	C29—H29B	0.9800
C7—C8	1.376 (4)	C29—H29C	0.9800
C7—H7	0.9500	C30—H30C	0.9800
C8—C9	1.399 (3)	C30—H30B	0.9800
C8—H8	0.9500	C30—H30A	0.9800
C9—C13	1.512 (3)	O1—C31	1.393 (5)
C10—C11	1.516 (4)	O1—C33B	1.416 (9)
C10—C12	1.519 (4)	O1—C33A	1.539 (9)
C10—H10	1.0000	C31—C32	1.462 (6)
C11—H11A	0.9800	C31—H31A	0.9900
C11—H11B	0.9800	C31—H31B	0.9900
C11—H11C	0.9800	C32—H32A	0.9800
C12—H12C	0.9800	C32—H32B	0.9800
C12—H12B	0.9800	C32—H32C	0.9800
C12—H12A	0.9800	C33A—C34A	1.473 (14)
C13—C14	1.525 (4)	C33A—H33A	0.9900
C13—C15	1.538 (4)	C33A—H33B	0.9900
C13—H13	1.0000	C33B—C34B	1.433 (14)
C14—H14A	0.9800	C33B—H33D	0.9900
C14—H14B	0.9800	C33B—H33E	0.9900
C14—H14C	0.9800	C34A—H34A	0.9800
C15—H15A	0.9800	C34A—H34B	0.9800
C15—H15B	0.9800	C34A—H34C	0.9800
C15—H15C	0.9800	C34B—H34D	0.9800
C16—H16	0.9500	C34B—H34E	0.9800
C17—C18	1.355 (3)	C34B—H34F	0.9800
C17—H17	0.9500		
			
N3^i^—Fe1—N3	180.00 (3)	C17—C18—N4	105.81 (17)
N3^i^—Fe1—N1	93.88 (6)	C17—C18—H18	127.1
N3—Fe1—N1	86.12 (6)	N4—C18—H18	127.1
N3^i^—Fe1—N1^i^	86.12 (6)	C20—C19—C24	123.7 (2)
N3—Fe1—N1^i^	93.88 (6)	C20—C19—N4	116.99 (18)
N1—Fe1—N1^i^	180.00 (6)	C24—C19—N4	119.3 (2)
N3^i^—Fe1—Br1	88.74 (4)	C21—C20—C19	116.8 (2)
N3—Fe1—Br1	91.26 (4)	C21—C20—C25	120.8 (2)
N1—Fe1—Br1	89.88 (4)	C19—C20—C25	122.40 (19)
N1^i^—Fe1—Br1	90.12 (4)	C22—C21—C20	120.9 (3)
N3^i^—Fe1—Br1^i^	91.26 (4)	C22—C21—H21	119.6
N3—Fe1—Br1^i^	88.74 (4)	C20—C21—H21	119.6
N1—Fe1—Br1^i^	90.12 (4)	C23—C22—C21	120.8 (2)
N1^i^—Fe1—Br1^i^	89.88 (4)	C23—C22—H22	119.6
Br1—Fe1—Br1^i^	180.0	C21—C22—H22	119.6
C1—N1—C2	105.58 (16)	C22—C23—C24	120.9 (2)
C1—N1—Fe1	125.49 (13)	C22—C23—H23	119.6
C2—N1—Fe1	128.27 (13)	C24—C23—H23	119.6
C1—N2—C3	106.83 (16)	C23—C24—C19	116.9 (2)
C1—N2—C4	126.10 (16)	C23—C24—C28	121.1 (2)
C3—N2—C4	127.01 (16)	C19—C24—C28	122.0 (2)
C16—N3—C17	105.29 (16)	C20—C25—C27	111.2 (2)
C16—N3—Fe1	123.77 (12)	C20—C25—C26	111.3 (2)
C17—N3—Fe1	127.33 (13)	C27—C25—C26	110.9 (2)
C16—N4—C18	107.06 (16)	C20—C25—H25	107.7
C16—N4—C19	125.48 (16)	C27—C25—H25	107.7
C18—N4—C19	126.45 (16)	C26—C25—H25	107.7
N1—C1—N2	111.41 (17)	C25—C26—H26A	109.5
N1—C1—H1	124.3	C25—C26—H26B	109.5
N2—C1—H1	124.3	H26A—C26—H26B	109.5
C3—C2—N1	109.73 (17)	C25—C26—H26C	109.5
C3—C2—H2	125.1	H26A—C26—H26C	109.5
N1—C2—H2	125.1	H26B—C26—H26C	109.5
C2—C3—N2	106.44 (18)	C25—C27—H27C	109.5
C2—C3—H3	126.8	C25—C27—H27B	109.5
N2—C3—H3	126.8	H27C—C27—H27B	109.5
C5—C4—C9	123.38 (18)	C25—C27—H27A	109.5
C5—C4—N2	117.93 (17)	H27C—C27—H27A	109.5
C9—C4—N2	118.68 (18)	H27B—C27—H27A	109.5
C6—C5—C4	117.0 (2)	C24—C28—C29	112.2 (2)
C6—C5—C10	120.6 (2)	C24—C28—C30	111.2 (3)
C4—C5—C10	122.40 (18)	C29—C28—C30	110.2 (2)
C7—C6—C5	120.8 (2)	C24—C28—H28	107.7
C7—C6—H6	119.6	C29—C28—H28	107.7
C5—C6—H6	119.6	C30—C28—H28	107.7
C6—C7—C8	121.4 (2)	C28—C29—H29A	109.5
C6—C7—H7	119.3	C28—C29—H29B	109.5
C8—C7—H7	119.3	H29A—C29—H29B	109.5
C7—C8—C9	120.5 (2)	C28—C29—H29C	109.5
C7—C8—H8	119.8	H29A—C29—H29C	109.5
C9—C8—H8	119.8	H29B—C29—H29C	109.5
C8—C9—C4	117.0 (2)	C28—C30—H30C	109.5
C8—C9—C13	120.7 (2)	C28—C30—H30B	109.5
C4—C9—C13	122.26 (19)	H30C—C30—H30B	109.5
C11—C10—C12	112.6 (3)	C28—C30—H30A	109.5
C11—C10—C5	110.5 (2)	H30C—C30—H30A	109.5
C12—C10—C5	112.1 (2)	H30B—C30—H30A	109.5
C11—C10—H10	107.1	C31—O1—C33B	98.5 (4)
C12—C10—H10	107.1	C31—O1—C33A	120.0 (4)
C5—C10—H10	107.1	O1—C31—C32	108.4 (3)
C10—C11—H11A	109.5	O1—C31—H31A	110.0
C10—C11—H11B	109.5	C32—C31—H31A	110.0
H11A—C11—H11B	109.5	O1—C31—H31B	110.0
C10—C11—H11C	109.5	C32—C31—H31B	110.0
H11A—C11—H11C	109.5	H31A—C31—H31B	108.4
H11B—C11—H11C	109.5	C31—C32—H32A	109.5
C10—C12—H12C	109.5	C31—C32—H32B	109.5
C10—C12—H12B	109.5	H32A—C32—H32B	109.5
H12C—C12—H12B	109.5	C31—C32—H32C	109.5
C10—C12—H12A	109.5	H32A—C32—H32C	109.5
H12C—C12—H12A	109.5	H32B—C32—H32C	109.5
H12B—C12—H12A	109.5	C34A—C33A—O1	107.1 (7)
C9—C13—C14	111.1 (2)	C34A—C33A—H33A	110.3
C9—C13—C15	112.4 (2)	O1—C33A—H33A	110.3
C14—C13—C15	110.0 (2)	C34A—C33A—H33B	110.3
C9—C13—H13	107.7	O1—C33A—H33B	110.3
C14—C13—H13	107.7	H33A—C33A—H33B	108.5
C15—C13—H13	107.7	O1—C33B—C34B	108.2 (6)
C13—C14—H14A	109.5	O1—C33B—H33D	110.1
C13—C14—H14B	109.5	C34B—C33B—H33D	110.1
H14A—C14—H14B	109.5	O1—C33B—H33E	110.1
C13—C14—H14C	109.5	C34B—C33B—H33E	110.1
H14A—C14—H14C	109.5	H33D—C33B—H33E	108.4
H14B—C14—H14C	109.5	C33A—C34A—H34A	109.5
C13—C15—H15A	109.5	C33A—C34A—H34B	109.5
C13—C15—H15B	109.5	H34A—C34A—H34B	109.5
H15A—C15—H15B	109.5	C33A—C34A—H34C	109.5
C13—C15—H15C	109.5	H34A—C34A—H34C	109.5
H15A—C15—H15C	109.5	H34B—C34A—H34C	109.5
H15B—C15—H15C	109.5	C33B—C34B—H34D	109.5
N3—C16—N4	111.78 (16)	C33B—C34B—H34E	109.5
N3—C16—H16	124.1	H34D—C34B—H34E	109.5
N4—C16—H16	124.1	C33B—C34B—H34F	109.5
C18—C17—N3	110.06 (18)	H34D—C34B—H34F	109.5
C18—C17—H17	125.0	H34E—C34B—H34F	109.5
N3—C17—H17	125.0		
			
C2—N1—C1—N2	−0.3 (2)	C19—N4—C16—N3	169.57 (18)
Fe1—N1—C1—N2	−171.58 (12)	C16—N3—C17—C18	0.0 (2)
C3—N2—C1—N1	0.2 (2)	Fe1—N3—C17—C18	158.93 (15)
C4—N2—C1—N1	177.60 (17)	N3—C17—C18—N4	0.3 (2)
C1—N1—C2—C3	0.2 (2)	C16—N4—C18—C17	−0.5 (2)
Fe1—N1—C2—C3	171.21 (14)	C19—N4—C18—C17	−169.40 (19)
N1—C2—C3—N2	−0.1 (2)	C16—N4—C19—C20	−74.2 (3)
C1—N2—C3—C2	−0.1 (2)	C18—N4—C19—C20	92.7 (2)
C4—N2—C3—C2	−177.42 (19)	C16—N4—C19—C24	106.0 (2)
C1—N2—C4—C5	−93.7 (2)	C18—N4—C19—C24	−87.0 (3)
C3—N2—C4—C5	83.2 (3)	C24—C19—C20—C21	−0.4 (3)
C1—N2—C4—C9	86.6 (2)	N4—C19—C20—C21	179.86 (19)
C3—N2—C4—C9	−96.5 (2)	C24—C19—C20—C25	179.7 (2)
C9—C4—C5—C6	1.2 (3)	N4—C19—C20—C25	0.0 (3)
N2—C4—C5—C6	−178.47 (18)	C19—C20—C21—C22	0.6 (4)
C9—C4—C5—C10	−177.9 (2)	C25—C20—C21—C22	−179.5 (2)
N2—C4—C5—C10	2.4 (3)	C20—C21—C22—C23	−0.5 (4)
C4—C5—C6—C7	−0.8 (3)	C21—C22—C23—C24	0.3 (4)
C10—C5—C6—C7	178.4 (2)	C22—C23—C24—C19	−0.1 (4)
C5—C6—C7—C8	−0.1 (4)	C22—C23—C24—C28	−179.8 (2)
C6—C7—C8—C9	0.6 (4)	C20—C19—C24—C23	0.2 (3)
C7—C8—C9—C4	−0.2 (3)	N4—C19—C24—C23	179.87 (19)
C7—C8—C9—C13	178.1 (2)	C20—C19—C24—C28	179.9 (2)
C5—C4—C9—C8	−0.8 (3)	N4—C19—C24—C28	−0.4 (3)
N2—C4—C9—C8	178.94 (18)	C21—C20—C25—C27	−63.4 (3)
C5—C4—C9—C13	−179.07 (19)	C19—C20—C25—C27	116.5 (2)
N2—C4—C9—C13	0.6 (3)	C21—C20—C25—C26	60.8 (3)
C6—C5—C10—C11	−73.6 (3)	C19—C20—C25—C26	−119.3 (2)
C4—C5—C10—C11	105.5 (3)	C23—C24—C28—C29	59.7 (3)
C6—C5—C10—C12	52.9 (3)	C19—C24—C28—C29	−120.0 (3)
C4—C5—C10—C12	−128.0 (3)	C23—C24—C28—C30	−64.2 (3)
C8—C9—C13—C14	−72.8 (3)	C19—C24—C28—C30	116.1 (3)
C4—C9—C13—C14	105.4 (3)	C33B—O1—C31—C32	−174.6 (6)
C8—C9—C13—C15	51.0 (3)	C33A—O1—C31—C32	165.9 (6)
C4—C9—C13—C15	−130.8 (2)	C31—O1—C33A—C34A	88.5 (8)
C17—N3—C16—N4	−0.3 (2)	C33B—O1—C33A—C34A	44.2 (12)
Fe1—N3—C16—N4	−160.22 (12)	C31—O1—C33B—C34B	171.7 (7)
C18—N4—C16—N3	0.5 (2)	C33A—O1—C33B—C34B	−46.0 (12)

Symmetry code: (i) −*x*+1, −*y*+2, −*z*+1.

### (IIb) trans-Dibromidotetrakis[1-(2,6-diisopropylphenyl)-1H-imidazole-κN3]iron(II) diethyl ether disolvate Hydrogen-bond geometry (Å, º)

Cg2 and Cg3 are the centroids of rings N3/N4/C16–C18 and C4–C9, respectively.

**Table:**

*D*—H···*A*	*D*—H	H···*A*	*D*···*A*	*D*—H···*A*
C1—H1···Br1^i^	0.95	2.76	3.399 (2)	125
C2—H2···Br1	0.95	2.89	3.479 (2)	121
C16—H16···Br1	0.95	2.86	3.4119 (18)	118
C17—H17···Br1^i^	0.95	3.02	3.542 (2)	116
C18—H18···O1^ii^	0.95	2.40	3.337 (3)	170
C15—H15*A*···*Cg*3^iii^	0.98	2.92	3.801 (3)	150
C25—H25···*Cg*2	1.00	2.61	3.413 (2)	137
C26—H26*A*···*Cg*3^iv^	0.98	2.87	3.682 (3)	140
C34*B*—H34*E*···*Cg*2^v^	0.98	2.92	3.627 (9)	130

Symmetry codes: (i) −*x*+1, −*y*+2, −*z*+1; (ii) −*x*+2, −*y*+2, −*z*+1; (iii) −*x*+1, −*y*+3, −*z*+1; (iv) −*x*+1, −*y*+2, −*z*+2; (v) *x*, *y*, *z*−4.
